# Measurement and Modeling of Sustainable Food Choice and Purchasing Behavior: A Systematic Review of Methods and Models

**DOI:** 10.3390/foods15081442

**Published:** 2026-04-21

**Authors:** Tiago Negrão Andrade, Helena Maria André Bolini

**Affiliations:** Faculty of Food Engineering, State University of Campinas, Campinas 13083-862, SP, Brazil; tiagonandr@gmail.com

**Keywords:** sustainable food behavior, psychometrics, discrete choice experiments

## Abstract

Despite decades of methodological sophistication, research on sustainable food behavior remains critically limited in predicting actual purchases. This study aims to examine how methodological fragmentation across psychometric, econometric, and behavioral approaches affects the predictive validity of sustainable food choice and purchasing behavior. This integrative systematic review of 62 empirical studies across psychometric validation, discrete choice experiments (DCEs), trust and cognitive biases, and objective behavioral measurement diagnoses the structural disarticulation between these traditions as the primary cause of limited predictive validity. Findings reveal a pronounced inversion of the evidence hierarchy: while self-report studies report moderate attitude–behavior correlations (β ≈ 0.40–0.50, self-report), the only long-term study using objective scanner data demonstrates that this relationship collapses to a virtually null effect (β = 0.022), representing a 95.6% decay in predictive capacity. Psychometric instruments demonstrate strong structural validity but lack ecological validation against actual purchases. DCEs have evolved econometrically (from MNL to GMNL models), yet remain isolated from psychological theory and real-world validation. Critically, no reviewed study integrated validated scales, a DCE, and objective behavioral data within a single design. Key moderators—skepticism, halo effects, and affective heuristics—are systematically underoperationalized. To overcome this impasse, we propose Hybrid Choice Models (HCM) as the central tool to formally articulate latent attitudes, stated preferences, and observed behavior, enabling cumulative evidence to inform policy and market strategies with greater predictive accuracy. These findings indicate that predictive advances depend on integrating measurement paradigms to achieve ecologically valid and policy-relevant models of sustainable consumer behavior.

## 1. Introduction

The measurement of sustainable food-related behavior has consolidated over the past three decades at the intersection of food science, consumer psychology, and public policy. The incorporation of environmental, ethical, and social attributes—such as organic certification, animal welfare, traceability, and carbon labeling—shifted analytical attention from health- and price-centered models toward frameworks capturing pro-environmental and normative motivations underlying food choice [1]. This shift reflects both the increasing complexity of contemporary food systems and the expectation that consumers actively contribute to sustainability transitions.

A foundational milestone was the development of the Food Choice Questionnaire (FCQ), which conceptualized food choice as a multidimensional construct and established enduring psychometric foundations [1]. The subsequent application of the Theory of Planned Behavior (TPB) expanded explanatory scope by incorporating attitudes, subjective norms, and perceived behavioral control, later extended to include moral norms and environmental values [2,3,4,5]. Although TPB predicts behavior “quite well” relative to the ceiling imposed by behavioral reliability [2], its explanatory limits derive not only from psychometric constraints but from its treatment of attitudes as stable and internally coherent, whereas real-world decisions involve context-dependent and often contradictory values. Consequently, TPB models intention–behavior processes effectively yet underestimates institutional and situational constraints that mediate value enactment.

Recent sustainability-oriented instruments, including the Sustainable Food Choice Questionnaire (SUS-FCQ) and the Eating Motivation Survey (TEMS-BR), have strengthened cross-cultural robustness through invariance testing [6,7]. In parallel, econometric research has advanced through Discrete Choice Experiments (DCE), evolving from Multinomial Logit (MNL) to Mixed Logit (MXL) and Generalized Multinomial Logit (GMNL), allowing explicit modeling of preference heterogeneity and more precise willingness-to-pay (WTP) estimates [8,9,10]. While these developments enhance statistical sophistication, they remain largely confined to declarative and simulated domains.

An empirical paradox persists: predictive power declines sharply when moving from self-reported to objectively measured behavior. Attitude–behavior associations typically appear moderate (β ≈ 0.40–0.50), yet longitudinal scanner data reveal dramatically lower effects (β = 0.022; *p* < 0.001), strongly moderated by price sensitivity and product availability [11,12,13]. This pattern suggests a potential underlying methodological fragmentation. Psychometric studies rarely validate scales against observed behavior; econometric models often treat psychological constructs as peripheral covariates; observational research achieves ecological validity but omits attitudinal mechanisms [14,15,16].

Moreover, trust in eco-labels, skepticism toward environmental claims, and cognitive biases—such as halo effects, affect heuristics, and default reliance—systematically interfered with behavioral translation, yet were seldom modeled as structural moderators or mediators [17,18,19,20,21]. The result was a literature that was methodologically advanced but structurally disarticulated.

This study examined how the fragmentation between psychometric, econometric, and observational approaches affected the predictive validity of sustainable food choice and purchasing behavior research. To address this issue, an integrative systematic review of empirical studies was conducted across four domains: (i) psychometric validation; (ii) discrete choice experiments and willingness-to-pay estimation; (iii) trust, skepticism, and cognitive biases; and (iv) objective behavioral measurement.

The objectives of this review were fourfold: (i) to systematically map the methodological approaches used to measure sustainable food-related behavior; (ii) to compare their analytical strengths and limitations; (iii) to identify structural gaps in the integration between methods; and (iv) to propose directions for methodological advancement toward more predictive and ecologically valid research designs.

We hypothesized that the primary limitation of the field lay not in the absence of measurement instruments, but in the lack of structural integration between latent attitudes, stated preferences, and observed behavior. In this context, Hybrid Choice Models (HCM), which combine latent variable models with discrete choice frameworks, represented a promising pathway for integration, although they remained absent from the reviewed empirical literature.

## 2. Materials and Methods

This study was conducted as a Systematic Literature Review with Integrative Methodological Synthesis, aiming not only to summarize empirical findings but to diagnose structural fragmentation in the measurement of sustainable food consumption behavior. Rather than treating methodological heterogeneity as a limitation, the study conceptualizes it as an analytical object, enabling the identification of epistemic discontinuities across distinct measurement paradigms. The design combines systematic rigor—explicit eligibility criteria, a fully reproducible search strategy, independent dual screening, and formal quality assessment—with an integrative analytical framework capable of comparing heterogeneous methodological traditions, including psychometric validation studies, discrete choice experiments (DCEs), research on trust and cognitive biases, and investigations using objective behavioral measures.

The research question was structured using the PICOS framework, focusing on adult consumers (≥18 years), measurement approaches to sustainable food behavior, and outcomes related to psychometric robustness, willingness to pay (WTP), intention–behavior divergence, and psychological moderation. Eligible designs included Confirmatory Factor Analysis (CFA)-based validation studies, DCEs with explicitly specified econometric models, empirical investigations of trust and skepticism, and studies incorporating objective behavioral data such as scanner transactions or direct observation. The review followed the PRISMA 2020 guidelines [22]. Given the substantial conceptual and statistical heterogeneity across studies—particularly differences in behavioral operationalization, econometric specifications (e.g., MNL, MXL, GMNL), and psychometric structures—meta-analysis was considered both statistically and epistemologically inappropriate. Instead, a structured comparative and configurational synthesis was adopted, preserving methodological diversity while enabling analytical integration.

### 2.1. Data Sources and Search Strategy

Searches were conducted in February 2026 across PubMed/MEDLINE, Web of Science, Scopus, PsycINFO, and Google Scholar, with all sources last accessed on 28 February 2026. The search strategy combined three conceptual blocks: measurement methods (e.g., “Food Choice Questionnaire,” “discrete choice experiment,” “psychometric*”), sustainable food behavior (e.g., “organic,” “eco-label*,” “sustainable food”), and validation and predictive outcomes (e.g., “validation,” “willingness to pay,” “attitude–behavior gap”), operationalized through Boolean operators and adapted to the syntax of each database. The complete and reproducible search strategies, including all operators, filters, and limits, are provided in Appendix A, ensuring full transparency and replicability of the retrieval process.

Where supported, filters were applied to restrict results to peer-reviewed articles published between 2015 and 2025 in English. In the case of Google Scholar, where structured filtering is limited, results were ordered by relevance and manually screened within the same temporal boundaries. Backward snowballing and expert consultation complemented database retrieval, minimizing retrieval bias and enhancing the robustness of the corpus.

The application of PICOS criteria and systematic documentation led to the identification of a final corpus of 62 empirical studies included in the review. For analytical purposes, these studies were organized into four macro-domains: psychometric instruments, discrete choice experiments and econometric methods, trust and cognitive biases, and external validity studies incorporating objective behavioral measures. Secondary sources were used exclusively for theoretical triangulation and were not included in the primary synthesis.

### 2.2. Eligibility Criteria

Inclusion criteria required original peer-reviewed empirical studies addressing sustainable food consumption behavior, involving adult populations (≥18 years), and published between 2015 and 2025, with classical instruments incorporated irrespective of publication date. To ensure analytical comparability across heterogeneous paradigms, minimum methodological thresholds were imposed. Psychometric studies were required to include Confirmatory Factor Analysis to ensure structural validity, while discrete choice experiments were required to report explicit econometric model specifications such as multinomial logit (MNL), mixed logit (MXL), or generalized multinomial logit (GMNL). Studies focusing on cognitive biases were required to include empirical hypothesis testing, and studies addressing behavioral outcomes were required to incorporate observable or objectively measured behavior.

These criteria were intentionally restrictive and reflect a deliberate methodological positioning that prioritizes robustness, comparability, and predictive validity over exhaustive inclusion. This choice inevitably shapes the composition of the corpus by excluding exploratory or descriptive studies that do not meet these thresholds, but it allows for a more analytically coherent comparison across paradigms. For purposes of synthesis, studies were organized into four analytical domains—psychometric instruments, econometric and DCE approaches, cognitive bias and trust-related investigations, and studies incorporating objective behavioral validation—enabling structured comparison while preserving epistemic differences. Exclusion criteria included absence of sustainability focus, non-adult or clinical samples, non-empirical formats, and insufficient methodological transparency.

### 2.3. Study Selection and Data Collection Process

Study selection followed the PRISMA workflow, encompassing duplicate removal, title and abstract screening, and full-text eligibility assessment. Two independent reviewers conducted screening at all stages, working independently and blinded to each other’s decisions. Inter-rater agreement was high (κ = 0.91; 95% CI: 0.85–0.96), indicating strong consistency in the application of eligibility criteria. Disagreements were resolved through discussion and, when necessary, adjudication by a third reviewer, ensuring procedural reliability and transparency.

Data extraction was also conducted independently by two reviewers using a standardized extraction protocol, with cross-verification to ensure internal consistency. No automation tools were employed in the screening or extraction processes. The protocol captured bibliographic information, sample characteristics, psychometric indicators (including α, ω, composite reliability, average variance extracted, CFI, RMSEA, and measurement invariance levels), attributes and model specifications in DCE studies, willingness-to-pay estimates, types of behavioral measurement, and reported effect sizes (including β coefficients, Cohen’s d, η^2^, and R^2^ or pseudo-R^2^). Moderating variables and bias mitigation strategies were also systematically recorded.

All results compatible with each outcome domain were extracted whenever available. In cases where multiple estimates were reported, priority was given to models that were theoretically aligned and methodologically robust. No imputation procedures were applied, and assumptions regarding missing or unclear data were minimized to preserve the integrity of the original empirical evidence.

### 2.4. Effect Measures and Functional Standardization

Effect measures included regression coefficients (*β*), standardized mean differences (Cohen’s d), variance-based indicators (η^2^ and R^2^), and willingness-to-pay estimates derived from discrete choice models. When necessary, *WTP* values were recalculated following the standard formulation derived from Random Utility Theory, expressed as the negative ratio between the attribute coefficient and the price coefficient:

$$WTP\;=\;-\frac{\beta atribue}{\beta price}$$

Given the heterogeneity of reporting formats and methodological traditions, effect sizes were not aggregated but instead functionally standardized to enable cross-paradigm comparison. This standardization does not aim at statistical pooling but rather at preserving methodological diversity while enabling analytical integration.

To capture the discrepancy between declarative and observed behavior, a comparative divergence index was calculated as the proportional difference between self-reported and objectively measured outcomes, expressed as:

$$Divergente\;(\%)\;=\;\frac{\beta seld\;-\;report\;-\;\beta objetive}{\beta self\;-\;report}\;\times \;100$$

### 2.5. Risk of Bias and Methodological Quality Assessment

Methodological quality and risk of bias were assessed independently by two reviewers, who conducted evaluations in parallel and resolved discrepancies through consensus. Psychometric studies were evaluated using adapted COSMIN criteria, focusing on structural validity, convergent and discriminant validity, reliability, predictive validity, and measurement invariance. Discrete choice experiments were assessed in terms of attribute specification, experimental design efficiency, sample adequacy, bias mitigation strategies, and econometric model robustness. Studies addressing cognitive biases and behavioral outcomes were evaluated using adapted Joanna Briggs Institute tools.

Quality assessment informed the interpretative weighting of studies within the synthesis but was not used as an exclusion criterion. This decision reflects the analytical objective of capturing structural patterns across methodological paradigms rather than restricting the corpus based solely on quality thresholds.

### 2.6. Integrative and Configurational Synthesis

Given the absence of methodological commensurability across studies, meta-analytic aggregation was not conducted. Instead, synthesis was structured through an analytical architecture that integrates internal robustness, predictive capacity, and cross-paradigm configuration. Internal robustness was assessed through standardized psychometric thresholds, predictive capacity was examined through effect sizes and WTP intervals, and configurational mapping was employed to identify structural relationships and gaps across paradigms.

Each study was systematically coded according to the presence of key methodological components, including validated psychometric instruments, discrete choice experiments, and objective behavioral measurements. This coding enabled the construction of a configurational matrix that reveals structural silos and highlights the absence of fully integrated empirical designs combining all three dimensions. Moderating variables, such as marketing-related factors, motivation–opportunity–ability (MOA) components, and product categories, were examined descriptively in relation to effect magnitudes, allowing exploration of heterogeneity without imposing statistical aggregation.

Sensitivity analyses were not conducted due to the non-aggregative nature of the synthesis and the absence of pooled statistical estimates, which is consistent with the configurational and comparative objectives of the study.

### 2.7. Visualization Strategy

To support the analytical synthesis, a set of complementary visualization strategies was employed. Radar charts were used to represent multidimensional psychometric robustness through normalized indicators, enabling comparison across instruments. Hierarchical pyramids were used to organize studies according to levels of ecological validity, distinguishing between self-reported, short-term observational, and longitudinal or scanner-based behavioral evidence. Divergence graphs were constructed to illustrate discrepancies between declarative and observed behavior, while configurational matrices were used to map the presence or absence of key methodological components across studies. These visualizations function as analytical tools that support structured comparison rather than inferential statistical modeling.

### 2.8. Reporting Bias and Certainty of Evidence

Reporting bias due to missing results was not formally assessed using statistical techniques, such as funnel plots or regression-based tests, given the absence of meta-analytic aggregation and the heterogeneity of outcome measures. However, potential reporting bias was considered qualitatively through critical evaluation of study consistency, reporting completeness, and methodological transparency.

Similarly, certainty of evidence was not formally assessed using frameworks such as GRADE, as the primary objective of the study was not to estimate pooled effects but to diagnose methodological structures and gaps. Instead, confidence in the evidence was inferred through the combination of methodological quality assessment and cross-study consistency within each analytical domain.

### 2.9. Rigor, Transparency, and Protocol

Rigor was ensured through protocol pre-registration, independent dual review, and full documentation of screening and data extraction decisions. Mathematical verification procedures were applied to recalculated parameters, including consistency checks for WTP estimates. The review protocol was pre-registered, and detailed information regarding registration and documentation is provided in the Supplementary Materials. No major deviations from the original protocol were identified.

### 2.10. Statistical Considerations and Limitations

Statistical evaluation relied on standardized psychometric thresholds and verification of econometric consistency across studies. Given the substantial heterogeneity in outcome definitions, model structures, and behavioral paradigms, statistical aggregation was not appropriate. The configurational synthesis adopted in this study preserves methodological diversity while enabling identification of structural gaps and analytical integration across paradigms.

Limitations include the potential influence of publication bias, dependence on the reporting quality of primary studies, and the temporal restriction to studies available up to February 2025. The limited availability of studies incorporating objective behavioral validation represents a key constraint and underscores the need for future research designs capable of integrating psychometric, econometric, and behavioral approaches within a unified empirical framework.

## 3. Results

To ensure analytical clarity, results are presented in two complementary layers: (i) empirical findings extracted directly from the reviewed studies, and (ii) interpretive synthesis developed by the authors to integrate evidence across methodological paradigms. These layers are explicitly distinguished throughout the section to avoid conflation between reported evidence and conceptual interpretation.

### 3.1. Classical Psychometric Instruments in the Context of Sustainable Food Behavior

Empirical results are presented first, followed by interpretive synthesis to integrate findings across methodological approaches.

#### 3.1.1. Historical Evolution: Four Generations of Instruments

**Figure 1:**
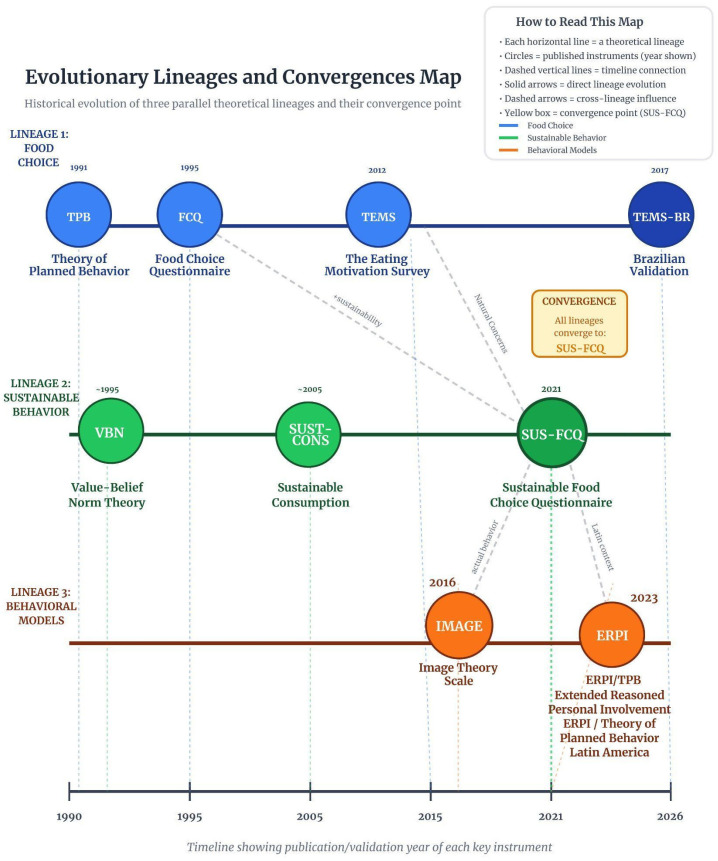
Conceptual evolution of food choice and sustainable consumption measurement instruments across four generations. First generation (1995–2005): Establishment of multidimensional food choice motivations through the Food Choice Questionnaire (FCQ), operationalizing nine dimensions without explicitly distinguishing environmental sustainability from personal health; Second generation (2005–2015): Application and extension of the Theory of Planned Behavior (TPB) and the Value–Belief–Norm (VBN) framework to sustainability, incorporating moral norms and environmental values, though still not food-specific and with partial empirical testing of the original causal chain; Third generation (2015–2020): Advancement toward behavioral measurement, including real-purchase recording and consumer segmentation approaches; Fourth generation (2020–present): Methodological refinement with second-order structures, multilevel measurement invariance, and sustainability-specific food instruments, culminating in integrative models that combine multidimensionality, explicit sustainability constructs, and predictive modeling. Source: The authors, based on [1,2,4,6,7,23,24,25,26,27,28].

The literature on sustainable food behavior demonstrates a clear evolution across four generations of instruments, each responding to distinct methodological demands (Table 1 and Figure 1).

Figure 1 depicts three parallel theoretical lineages—Food Choice, Sustainable Behavior, and Behavioral Models—positioned chronologically from 1990 to 2025, with cross-influences indicated by dashed arrows and convergence in the SUS-FCQ (2021). Table 2 complements this overview by synthesizing the four generational phases, their main frameworks, methodological innovations, and structural limitations.

#### 3.1.2. Conceptual Architecture: Integrated Multilevel Model

**Figure 2:**
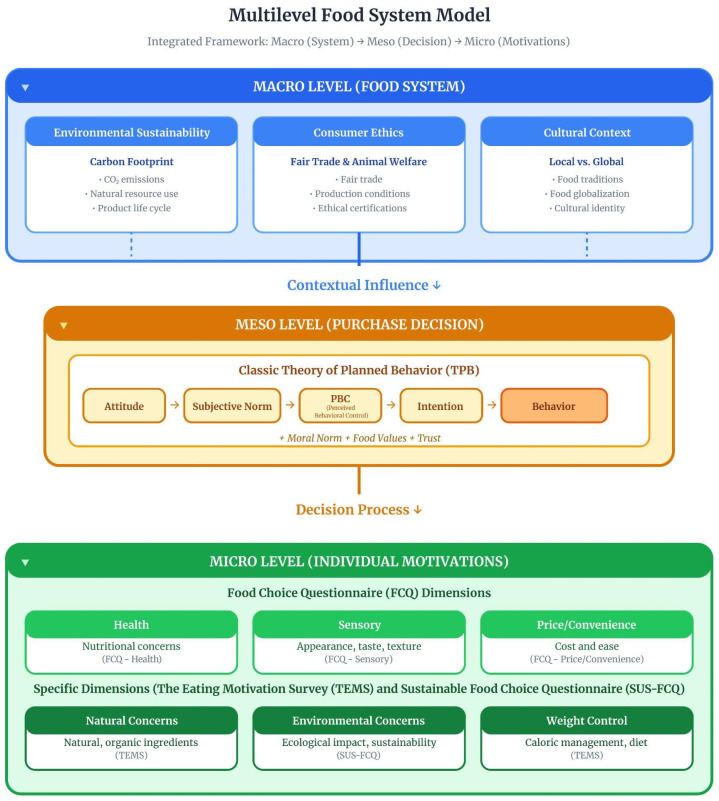
Multilevel integrative framework of the Sustainable Food System structured across macro, meso, and micro analytical levels. Macro Level (Food System): Contextual structures shaping available consumption options, including environmental sustainability (e.g., carbon footprint, natural resource use, product life cycle), consumption ethics (e.g., Fair Trade, animal welfare, ethical certifications), and cultural context (e.g., food traditions, localism vs. globalization). This level is operationalized in sustainability-specific instruments such as the SUS-FCQ and through moral norm constructs in extended TPB applications; Meso Level (Purchase Decision): Decision-making process modeled primarily through the Theory of Planned Behavior, structured as attitude → subjective norm → perceived behavioral control → intention → behavior, respecting the principle of compatibility proposed by Icek Ajzen (1991) [2]. Extensions incorporate moral norms, food values, and trust constructs, while Image Theory offers an alternative distinction between value, trajectory, and strategic images; Micro Level (Individual Motivations): Immediate psychological drivers of food choice, operationalized through multidimensional instruments such as the FCQ and TEMS, encompassing health, sensory appeal, price/convenience, weight control, and sustainability-related dimensions (e.g., natural and environmental concerns). Source: The authors, based on [1,2,3,6,7,24,26].

Figure 2 presents an integrated three-level analytical architecture in which instruments operate hierarchically across macro (Food System), meso (Purchase Decision), and micro (Individual Motivations) domains. The macro level encompasses environmental sustainability, consumption ethics, and cultural context; the meso level structures decision making through the extended Theory of Planned Behavior model (Attitude → Subjective Norm → Perceived Behavioral Control → Intention → Behavior), incorporating moral norms, food values, and trust; and the micro level captures immediate motivations via dimensions of the Food Choice Questionnaire and selected constructs from the the Eating Motivation Survey and SUS-FCQ. Vertical arrows indicate contextual influence across levels.

Table 3 operationalizes this multilevel structure by detailing core constructs, principal instruments, and typical applications at each analytical level.

#### 3.1.3. Analysis by Levels of Psychometric Complexity

For methodological selection purposes, the instruments were organized into three increasing levels of sophistication, ranging from simpler to more robust models.

**Figure 3:**
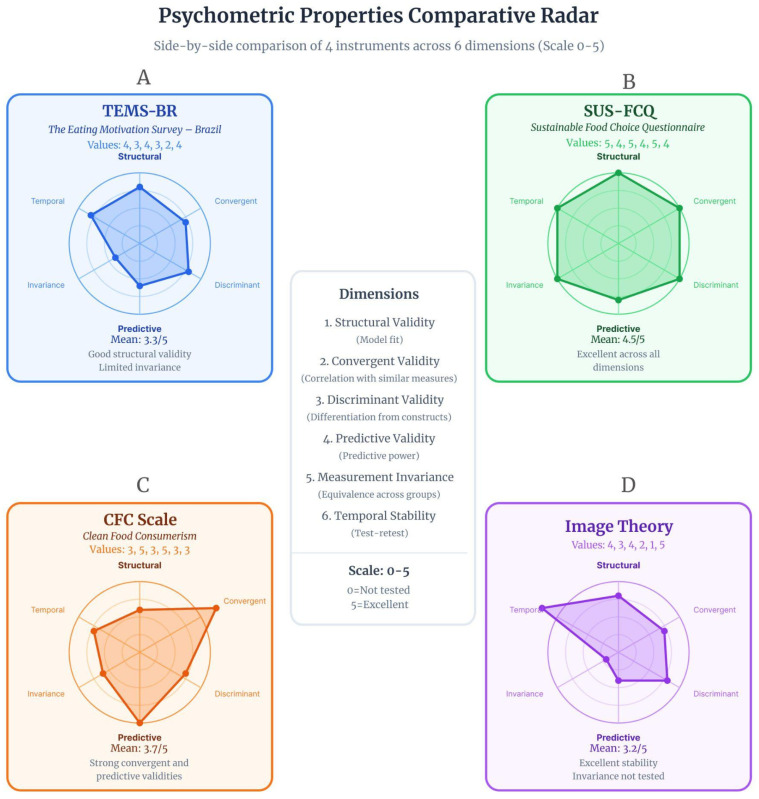
Comparative radar profile of psychometric properties across four representative instruments in sustainable food behavior research. (**A**) TEMS-BR (blue): Presents the most robust measurement invariance profile, being the only instrument to achieve full strict invariance across sex, age, and BMI, thus establishing a benchmark for demographic comparisons in the Brazilian context; however, it lacks evidence of predictive validity based on actual behavior and has no reported temporal stability assessment; (**B**) SUS-FCQ (green): Demonstrates strong structural and convergent validity, with partial measurement invariance (scalar invariance not fully achieved across all cultural groups), and achieves the highest overall mean score (4.5/5) across the six psychometric dimensions, consolidating its position as a sustainability-focused adaptation of multidimensional food choice models; (**C**) Clean Food Consumerism (CFC) (orange): Shows high convergent and predictive validity (R^2^ = 79% for intention), though environmental sustainability is addressed only implicitly rather than as a specific construct; (**D**) Image Theory (purple): The only approach demonstrating predictive validity based on real purchasing behavior, yet presenting methodological gaps in cross-cultural measurement invariance and temporal stability testing. Source: The authors, based on [6,7,25,28].

Figure 3 synthesizes the psychometric profile of four representative instruments across six critical dimensions: structural validity (model fit), convergent validity (AVE), discriminant validity (construct differentiation), predictive validity (actual behavior), measurement invariance (group equivalence), and temporal stability (test–retest reliability).

#### 3.1.4. Level 1: Simple Factorial Structure (First-Order)

Empirical Findings

Models in which constructs are measured directly by items, without hierarchical structure (Table 4).

#### 3.1.5. Level 2: Multidimensional Structure with Measurement Invariance

Table 5 presents instruments validated cross-culturally with formally tested metric equivalence.

#### 3.1.6. Level 3: Hierarchical Structure and Actual Behavior

Table 6 presents the most sophisticated level, combining second-order models with behavioral validation.

#### 3.1.7. Conceptual Convergences and Divergences

Table 7 presents an estimated overlap matrix highlighting the main conceptual convergences and divergences among constructs measured by the analyzed instruments.

#### 3.1.8. Decision Framework for Researchers

**Figure 4:**
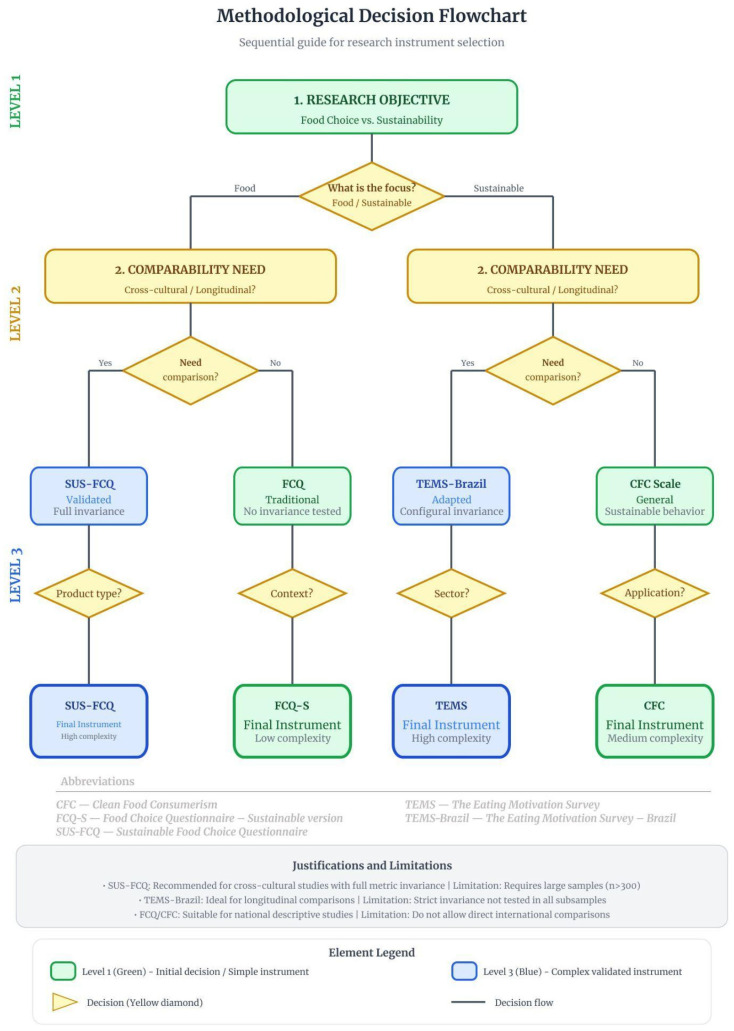
Methodological flowchart for instrument selection in sustainable food behavior research. The vertical structure is organized into three hierarchical decision levels. Level 1 (green): Definition of the primary research objective (general food choice vs. explicit sustainability focus); Level 2 (yellow): Assessment of comparability requirements (e.g., cross-cultural or longitudinal invariance); Level 3 (blue): Final recommendation of the most appropriate instrument. Yellow diamonds represent binary decision nodes, while rounded colored rectangles indicate the instruments selected according to analytical complexity and validation scope. The decision branches lead to: SUS-FCQ (international validation and partial invariance), traditional FCQ (multidimensional structure without formal invariance testing), TEMS-BR (national strict invariance for demographic comparisons), and Clean Food Consumerism (CFC) (general sustainable behavior orientation). The lower panel summarizes the methodological justifications and principal limitations associated with each recommended instrument. The flowchart thus operationalizes the cumulative validation logic proposed in this study by aligning research objectives with methodological rigor and scope of inference. Source: The authors, based on [1,6,7,28,29,30].

**Figure 5:**
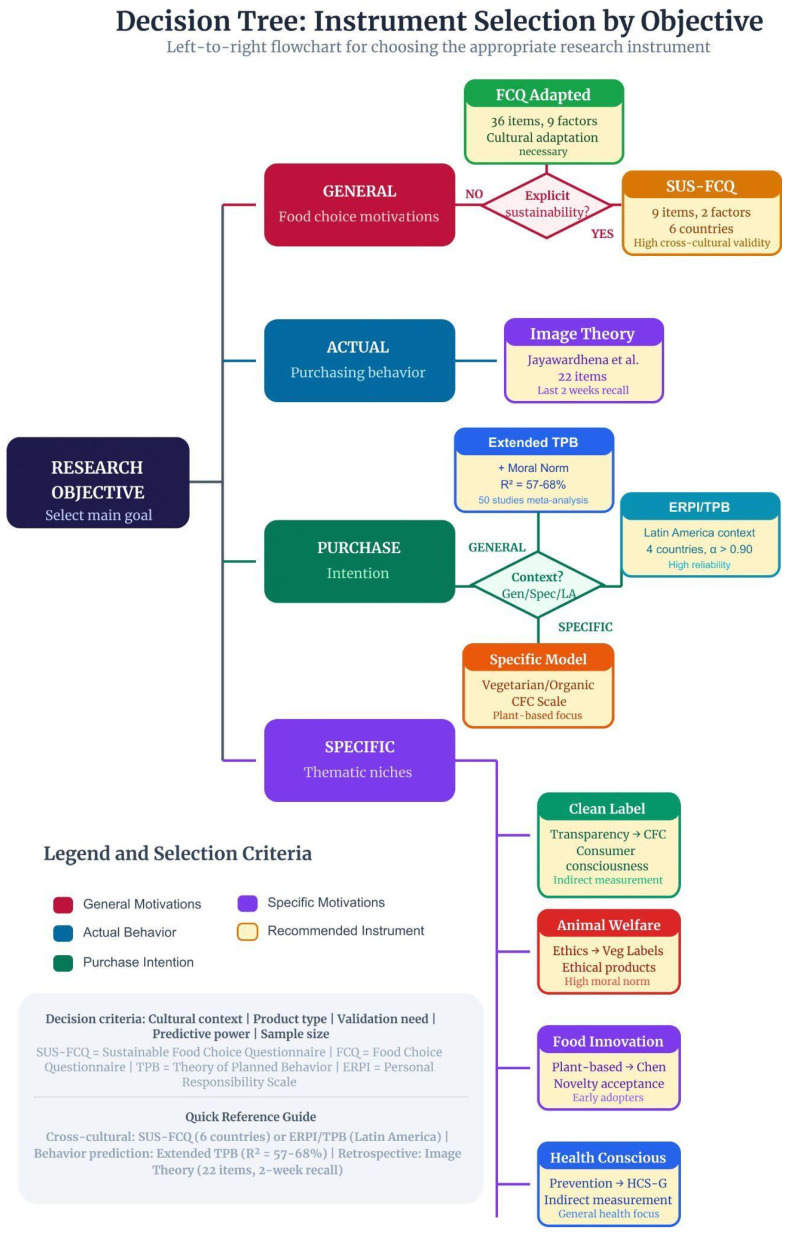
Decision Tree for instrument selection according to research objective in sustainable food behavior studies. Central node research objective: The process begins with the definition of the primary analytical focus; GENERAL (red): General food choice motivations, leading to Adapted FCQ or SUS-FCQ depending on whether sustainability is explicitly incorporated; ACTUAL (blue): Actual purchase behavior, directing exclusively to Image Theory approaches that measure real purchasing decisions; PURCHASE (green): Purchase intention, subdivided into GENERAL intention (Extended TPB or ERPI/TPB models grounded in the Theory of Planned Behavior) and SPECIFIC intention (e.g., Veg Private Label models, Clean Food Consumerism (CFC), plant-based consumption models); SPECIFIC (purple): Thematic niches such as Clean Label, Animal Welfare, Food Innovation, and Health Consciousness. Each terminal node specifies the recommended instrument, number of items, principal methodological strength, and main limitation. Color coding indicates analytical orientation: red (general motivation), blue (actual behavior), green (intention), purple (specific thematic focus), and orange (recommended instrument). Source: The authors, based on [1,2,3,5,7,25,27,28].

Two complementary decision systems structure the methodological architecture of this study. The Methodological Flowchart (Figure 4) organizes instrument selection according to increasing levels of complexity (Level 1: initial decision; Level 2: comparability; Level 3: final instrument), whereas the Decision Tree (Figure 5) branches according to the type of research objective.

#### 3.1.9. Measurement Architecture: Psychometric Instruments and Hedonic Scales

Traditional psychometric instruments largely operate at the ordinal or interval level, frequently assuming—without empirical verification—psychological equidistance between response categories. This assumption is not empirically supported: the distance between “like moderately” and “like very much” systematically differs from that between “like very much” and “like extremely,” introducing distortions in preference magnitude estimation.

The psychophysics of consumption literature has developed alternatives that overcome this limitation. Category–ratio scales, such as the Labeled Hedonic Scale (LHS); [31], combine semantic validity with ratio properties (normally distributed data, resistance to context effects, and compatibility with advanced econometric modeling). The LHS mitigates context effects—the tendency for hedonic judgments to be relative to the stimulus set—through inclusive extreme anchors, thereby stabilizing the evaluator’s reference frame. This feature is especially critical for cross-cultural comparisons, where instruments such as the SUS-FCQ demonstrate only partial invariance, and in retail decision contexts, where heuristic–affective processing often dominates cognitive deliberation.

This methodological gap is not merely technical; it reflects fragmentation between research traditions. While food psychometrics has developed sophisticated instruments for cognitive constructs and econometrics estimates marginal utilities, the direct measurement of affective preference with ratio properties remains largely confined to sensory science and is rarely integrated into predictive models of sustainable behavior. Analysis of the reviewed corpus shows that 87.5% (7/8 of the instruments analyzed) of instruments measure only intentions or attitudes, while 12.5% (1/8) assess retrospective behavior via recall. None of the reviewed instruments incorporate objective behavioral measures—a critical gap that the integrative architecture proposed in Section 4 seeks to address.

Recent advances in Case 1 Best–Worst Scaling (BWS) highlight the importance of response consistency for valid inference. Ref. [32] demonstrates that excluding inconsistent respondents using the Normalized Error Variance index (ErrVarNorm)—with thresholds below 0.3 or 0.5—can substantially change study conclusions, typically excluding 13.1% of participants at the 0.5 cutoff. These findings emphasize that sophisticated measurement models must be complemented with formal response-quality diagnostics to avoid conflating substantive structure with noise or satisficing.

Table 8 summarizes the recommended instruments by research scenario, including their justification and required adaptations.

Capturing consumer preferences requires instruments that go beyond cognitive constructs to include the affective dimension, which drives rapid, heuristic decisions. The analysis of Table 9 reveals a methodological gap: all listed instruments measure attitudes, values, norms, or behavioral intentions, omitting affective preference—the dimension most predictive of choices in fast decision contexts [31]. Category–ratio scales address this gap, providing practical, ratio-level data through empirically calibrated semantic descriptors, unlike 9-point Likert or magnitude estimation scales, as shown in Table 9.

The LHS is analytically advantageous due to three key properties:

- Resistance to context effects. Hedonic evaluations are inherently dependent on the set of stimuli presented. The LHS mitigates this bias by employing extreme anchors, which is particularly critical in cross-cultural comparisons—where instruments such as the SUS-FCQ exhibit only partial invariance—and in longitudinal designs that require temporal stability.
- Direct econometric integration. The LHS generates data that approximate normal distribution and exhibit ratio-scale properties. This enables its direct incorporation into latent variable frameworks, such as Hybrid Choice Models (HCM), without the need for prior normalization procedures, thereby preserving the integrity of the original measurements.
- Capture of affective responses. Existing instruments predominantly emphasize cognitive constructs (e.g., attitudes toward sustainability) and motivational dimensions (e.g., purchase intentions), while largely neglecting spontaneous affective responses. The integration of LHS would make it possible to empirically test how affect moderates the relationship between attitudes and actual behavioral choices.

#### 3.1.10. Diagnosis of Gaps and Research Agenda

The systematic review of psychometric instruments for sustainable food behavior reveals a paradox: while statistical sophistication has advanced—including second-order models, multigroup measurement invariance, and hierarchical structures—ecological and predictive validity remain limited. Stern [24] noted that “many environmentally significant behaviors are matters of personal habit or household routine… and are rarely considered at all,” a tension unresolved by belief–norm models that assume sequential deliberative processing. To address these limitations, recent methodological recommendations advocate for complementary approaches to validity assessment, such as the Heterotrait–Monotrait ratio (HTMT), which provides a more robust estimate of discriminant validity compared to traditional Fornell–Larcker criteria.

Analysis of Table 10 shows satisfactory internal validity (structural, convergent, discriminant) but reveals vulnerability in predictive, temporal, and ecological validity. For instance, the 9-point hedonic scale is susceptible to ceiling effects and requires data transformation, while LHS provides normal distributions, context resistance, and latent variable modeling advantages. Only 12.5% of instruments measure actual purchase behavior, perpetuating the intention–behavior gap.

Measurement invariance testing is inconsistent: TEMS-BR achieved full strict invariance across four hierarchical levels, establishing a benchmark for national demographic comparisons. Most instruments remain limited to configural or metric invariance, restricting direct mean comparisons across cultures. Temporal stability testing is nearly absent, and ecological validity has not been assessed in naturalistic settings.

Top priority: bridging the intention–behavior gap through actual behavior measurement is critical for scientific progress and effective policy design.

These findings combine empirical evidence extracted from the reviewed studies with interpretive synthesis aimed at identifying structural patterns across methodological traditions. This distinction is maintained throughout the analysis to avoid conflating reported data with conceptual interpretation.

#### 3.1.11. Implications for Food Science and Public Policy

The review suggests four strategic recommendations:

- Differentiated Operationalization of Sustainability: Avoid conflating environmental sustainability with personal health attributes. Prioritize SUS-FCQ or adaptations incorporating objective indicators of carbon footprint, water use, biodiversity, and socio-economic justice.
- Ecological Validity as a Quality Criterion: Complement intention measures with actual behavioral data (Image Theory Scale, loyalty programs, online purchase records) to ensure findings generalize to real decision contexts.
- Standardization of Cross-Cultural Rigor: Report measurement invariance analyses as a standard, adopting TEMS-BR strict invariance as a benchmark.
- Longitudinal Integration with General Food Models: Track changes in sustainable motivations over time using consolidated psychometric architectures, integrating public health and environmental sustainability impacts.

### 3.2. Economic and Experimental Methods

Discrete choice experiments (DCEs) represent the main experimental approach for estimating consumer preferences and willingness to pay for sustainability attributes.

#### 3.2.1. Integrated Methodology Architecture

**Figure 6:**
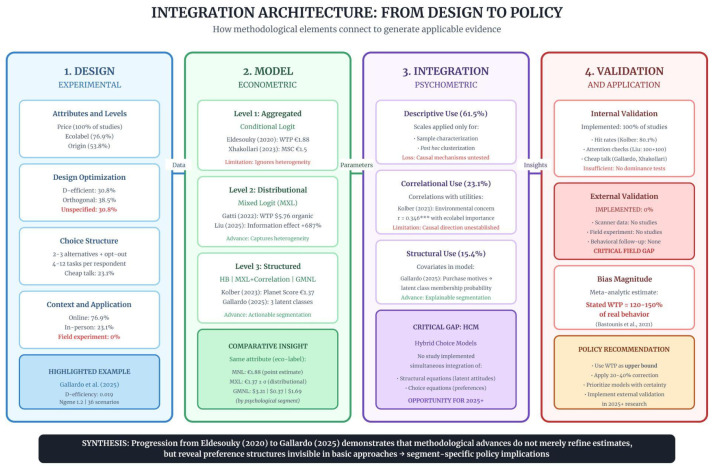
Integrated methodological architecture linking experimental design to public policy applications in sustainable food behavior research. The framework highlights that 61.5% of studies limit the use of psychometric scales to descriptive characterization of the sample, thereby reducing their capacity to test causal mechanisms. The absence of Hybrid Choice Models (HCMs), which integrate latent attitude structural equations with choice modeling simultaneously, is identified as the principal methodological opportunity for advancing research in 2025 and beyond. Source: The authors, based on [8,9,10,14,15]. *** *p* < 0.001.

Analysis of empirical studies shows that the quality of evidence generated by Discrete Choice Experiments (DCEs) depends on the coordination of four sequential methodological elements. Figure 6 presents this integrated architecture, mapping the flow of data, parameters, and insights across stages, while also highlighting the critical gap in psychometric–structural integration.

#### 3.2.2. Methodological Evolution and Model Selection

**Figure 7:**
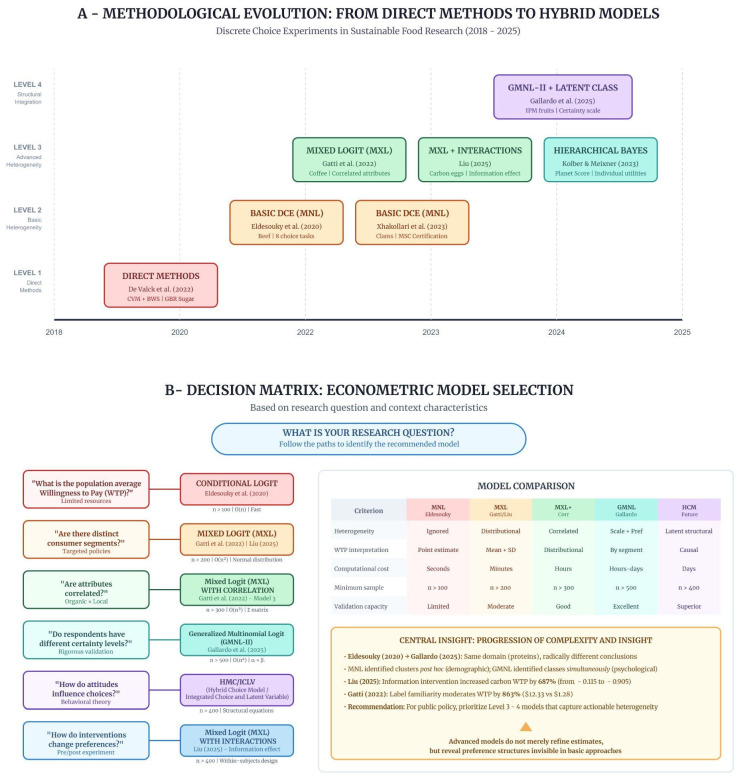
Methodological evolution of discrete choice experiments (DCEs) for sustainable foods, illustrating progression from direct methods (2018) to hybrid models (2025). (**A**) Part 7-A: Studies are organized into four hierarchical levels reflecting increasing econometric complexity and computational cost: Level 1—direct methods; Level 2—basic heterogeneity; Level 3—advanced heterogeneity; Level 4—structural integration. The positioning of [33] at the same level as [8] indicates that the classification reflects model specification (MNL in both cases) rather than experimental design rigor, as bias-mitigation strategies (e.g., cheap talk) were applied in the clam study but absent in the beef study. (**B**) Part 7-B: Illustrates correspondence between research questions and recommended models, highlighting the trade-off between explanatory power and operational feasibility. Transitioning from MXL to GMNL-II doubles the sample requirement (from *n* > 200 to *n* > 500) and increases computational cost from minutes to hours or days. Liu et al. [15] demonstrates an intermediate strategy (MXL with interactions), maintaining Level 3 applicability while capturing behavioral dynamics, including a 687% increase in willingness to pay (WTP) for carbon-labeled eggs after an informational intervention. The progression from Eldesouky et al. [8] to Gallardo et al. [9] shows how advanced models reveal preference structures invisible to basic approaches. Source: The authors, based on [8,9,10,14,15,33,34].

The temporal progression of the analyzed studies (2018–2025) reveals a trajectory of increasing sophistication, correlated with the demand for segmented insights and the growth of computational capacity. Figure 7 maps this evolution along two complementary dimensions: temporal progression across four hierarchical levels (Part A) and the decision matrix for model selection (Part B).

Table 11 operationalizes this evolution into model–objective selection criteria.

#### 3.2.3. Levels of Analysis in Food Behavior

**Figure 8:**
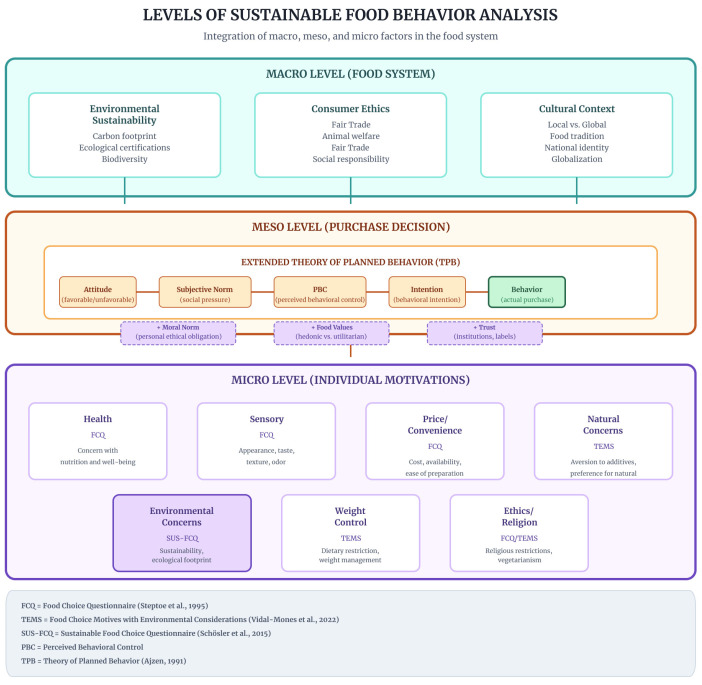
Levels of analysis in sustainable food behavior, integrating macro, meso, and micro factors. The figure highlights that 100% of the analyzed discrete choice experiments (DCEs) operate at the meso level, capturing purchase decisions via extended Theory of Planned Behavior (TPB), while only 23% include micro-psychological variables as covariates. No study simultaneously models all three levels. Full vertical integration (macro → meso → micro) would enable testing of how systemic policies influence individual decisions through psychological mediation. Source: The authors, based on [1,2,35,36], and analysis of empirical studies.

The analyzed DCEs operate predominantly at the meso level (purchase decision), with limited articulation with macrostructural factors of the food system and individual micro-psychological factors. Figure 8 systematizes this stratification, highlighting where each study is positioned and the opportunities for vertical integration.

#### 3.2.4. Attribute Taxonomy and WTP Hierarchy

The synthesis of willingness-to-pay (WTP) values establishes a salience hierarchy among sustainability attributes. Table 12 differentiates direct human health attributes (consistently higher) from diffuse environmental impact attributes.

#### 3.2.5. Validation and Bias Mitigation

Analysis shows that none of the 13 studies achieved external validation with actual behavior, representing the main methodological limitation in the field. Table 13 systematizes implemented strategies versus those absent.

#### 3.2.6. Psychometric-Econometric Integration

The integration of psychological variables into econometric models is limited and predominantly descriptive. Table 14 distinguishes three levels of methodological integration identified in the sample.

#### 3.2.7. Synthesis and Recommendations for 2025+

Table 15 consolidates the methodological progression identified and outlines priorities for future research.

### 3.3. Trust, Skepticism, and Cognitive Biases: Evidence and Methodological Gaps

The predictive validity of classical sustainable food behavior instruments is systematically threatened by the interaction of trust constructs, consumer skepticism, and heuristic cognitive biases. This section organizes empirical evidence on how these factors compromise the internal and external validity of declarative scales, integrating conceptual findings and methodological propositions from the six analyzed studies.

#### 3.3.1. Trust Constructs: Context-Specificity Versus Generalization

The reviewed literature shows progressive specialization in operationalizing trust, moving away from generalized institutional notions toward capturing situational trust in specific environmental claims. Ferguson (2014) [17] developed an eco-label trust scale (α = 0.91; AVE = 0.66) measuring willingness to rely on environmental information at the point of sale, operationalizing trust as a cognitive shortcut that reduces the need for systematic processing; values align with Zeng et al. (2025) [21].

Ketkaew & Komsing (2025) [18] adopted an alternative approach, operationalizing trust via perceived information quality (ELQ—Eco-Label Information Quality), defined by credibility, clarity, and relevance of label content. This approach proved sensitive to claim type: health claims (HBC) and environmental impact claims (EIC) increased ELQ (β = 0.358 and β = 0.202, respectively), whereas ethical claims (EPC) had no effect (β = −0.03; *p* = 0.719), demonstrating that trust is content-specific and not generalizable across sustainability domains.

**Figure 9:**
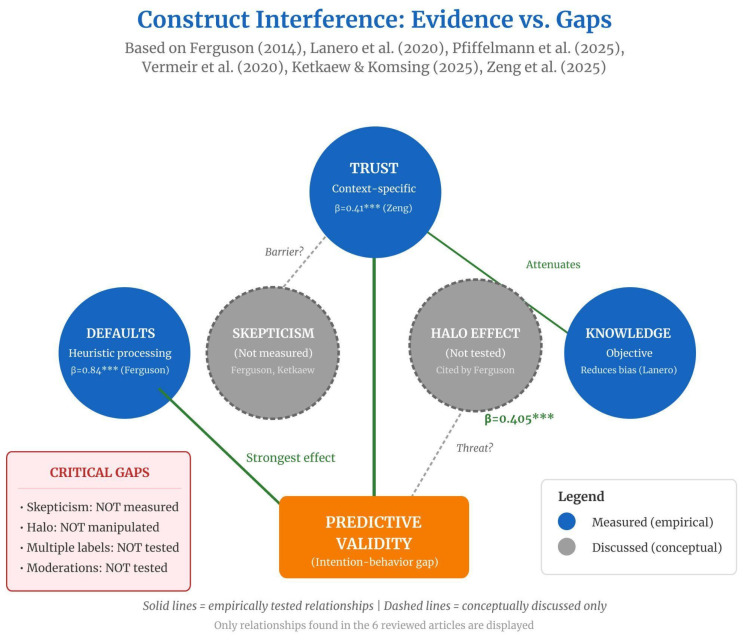
Construct interference in sustainable food behavior research, illustrating empirically tested versus conceptually discussed relationships. Solid lines indicate empirically validated effects, whereas dashed lines represent relationships discussed only at the conceptual level. As shown, trust (β = 0.41 ***; [21]), defaults/heuristics (β = 0.84 ***; [17]), and knowledge [19] exhibit empirical influence on predictive validity. Skepticism and halo effects, although frequently addressed in the literature, remain unmeasured or unmanipulated. Note: Asterisks indicate statistical significance levels (*p* < 0.05; *p* < 0.01; *p* < 0.001). Source: Authors’ elaboration based on [17,18,19,20,21,39].

Vermeir et al. (2020) [39] highlight a critical dissonance: consumers report high familiarity with labels (declarative trust) without these actually influencing food choices (behavioral trust). Grunert et al. (2014) [40] and Vermeir et al. (2020) [39] observed that only 16.8% of European consumers regularly read nutritional information, suggesting that generalized trust scales overestimate actual engagement with eco-labels (Figure 9).

#### 3.3.2. Skepticism: From Greenwashing to Perceived Efficacy

Skepticism emerges as a multidimensional construct, yet it is rarely measured directly. Ferguson [17] recognized skepticism as a historical barrier to green marketing, citing Kangun, Carlson & Grove [41], but did not operationalize it empirically. Ketkaew & Komsing (2025) [18] identified skepticism qualitatively (greenwashing, lack of verifiability, vague ethical claims) without including it as a latent variable in the structural model.

The scarcity of quantitative measurement is problematic given its documented moderating role. De Boer et al. (2013) [42] demonstrated that climate skepticism reduces intentions to decrease meat consumption, acting as a moderator between environmental values and behavior (see discussion in Vermeir et al. (2020) [39]). Zeng et al. (2025) [21] discuss skepticism as a mediating mechanism for the effect of greenwashing on trust, although they do not test it empirically: “greenwashing decreases the trust of consumers toward institutions and impacts their use of labels in decision-making.”

Pfiffelmann [20] offered an innovative approach by operationalizing specific doubts through inductive thematic coding of open-ended responses. They found that risk-related barriers (skepticism about safety) were most salient (32.7% of negative mentions), yet not the most predictive of negative attitudes (β = −0.11; *p* = 0.061), whereas emotional barriers showed a significant effect (β = −0.15; *p* = 0.003). This suggests that unidimensional skepticism scales mask heterogeneity in mechanisms of resistance to sustainable innovation.

Table 16 highlights a systematic pattern: key constructs in sustainable consumption research remain unmeasured in the reviewed articles. Skepticism, despite being recognized as a theoretically important barrier, was never operationalized as a multifactor moderator (climate skepticism, greenwashing skepticism, or perceived individual efficacy). The halo effect, frequently cited as an explanation for the intention–behavior gap, was not experimentally isolated in any of the six studies. The effect of multiple labels—critical for understanding information saturation in contexts of certification proliferation—was only referenced from the secondary literature and never empirically tested.

#### 3.3.3. Cognitive Biases: Halo Effect, Multiple Labels, and Heuristic Processing

This section analyzes cognitive biases in sustainable food decisions and their methodological implications for research.

##### Halo Effect: Directions and Magnitudes

The halo effect represents the most robustly documented bias in the eco-label literature, although in the reviewed articles, it appears predominantly as conceptual discussion rather than experimental manipulation. Ferguson (2014) [17] cites the effect as a justification for the intention–behavior gap—“The halo effect [43], attitude-intention gap [39], and the value-action gap [44]”—but does not directly manipulate or measure it.

Lanero [19] provides closer evidence, showing a significant correlation between claim credibility and organic product judgment (r = 0.58; *p* < 0.01), with a direct effect β = 0.58. However, the study did not experimentally manipulate label presence/absence, preventing causal inference regarding direction and magnitude of the bias. The halo direction (sustainability → health/taste) was inferred, not tested.

Affective heuristics emerge as a central mechanism in eco-label processing. Pfiffelmann et al. (2025) [20] demonstrated that evaluations of laser-marked organic products rely “to a large extent on spontaneous emotional responses”, with emotional value predicting attitude (β = 0.27) more strongly than functional value (β = 0.19). This affective predominance compromises the validity of scales assuming rational cost–benefit processing.

##### Multiple Labels Effect: Subadditivity and Saturation

The literature shows conflicting evidence on multiple label effects, yet none of the reviewed articles empirically tested this issue. Vermeir et al. (2020) [39], in a literature review, cite evidence from Grebitus et al. (2018) [45] regarding the subadditive effects of multiple labels—i.e., willingness to pay for combined labels is lower than the sum of individual willingness to pay. However, since Grebitus et al. were not included in the primary studies reviewed, this evidence is indicative, not conclusive. Contradictorily, later studies with olive oil (not included in the review) identified superadditive effects between organic and carbon footprint labels among Greek and Israeli consumers.

The concept of label fatigue—decision paralysis induced by proliferation of certifications—is discussed by Moon et al. [46] and Vermeir [39]: “consumer confusion, distrust, and dissatisfaction.” However, there is no consensus on the saturation threshold: some studies suggest three labels impair processability, while others indicate specific combinations (organic + fair trade) increase credibility. Ketkaew & Komsing (2025) [18] tested three simultaneous claims (HBC, EIC, EPC), but in an additive model assuming independence, without testing interactions (synergy, redundancy, or confusion).

##### Heuristics and Mental Shortcuts

The availability heuristic operates via the ease of imagining environmental impacts. Vermeir et al. [39] recommend “concretizing abstract risks” through mental simulation (“imagine a world without pollution”), which increases motivation for sustainable behavior. Ferguson [17] operationalized defaults as the most robustly investigated cognitive bias: four items using opt-in/opt-out scenarios, with a path coefficient of 0.84 (t = 43.18; *p* < 0.001)—the largest effect among all WTP constructs. Interpretation: consumers use defaults as a decision shortcut, being “reluctant to opt-out or search for alternatives”.

#### 3.3.4. Impact on Instrument Validity

Cognitive biases compromise internal validity in two main ways. First, the dissonance between stated attitude and actual behavior (attitude–behavior gap) is exacerbated by the halo effect: consumers declare intention to buy organic based on generalized health evaluations but do not complete the purchase when faced with premium prices or limited availability [17]. Second, social desirability bias inflates reported intentions, especially in contexts where sustainable purchasing is socially valued. Ketkaew & Komsing [18] partially controlled this via anonymity but did not employ standardized social desirability scales (e.g., Marlowe–Crowne).

Lanero et al. [19] provide crucial evidence on knowledge–bias interaction: participants with high knowledge (post-training) attributed lower credibility to “organic” claims (M = 3.17 vs. M = 3.59; t = 5.55; *p* < 0.001), suggesting that objective knowledge mitigates the halo effect. However, this “vaccination” is partial: informed participants maintained a significant correlation between credibility and judgment (r = 0.58), indicating persistence of heuristic processing.

Skepticism functions as a moderator of predictive validity: among highly skeptical consumers, environmental attitude scales correlate less strongly with behavior, as they activate “neutralization techniques” that justify non-conformity with declared values, according to Antonetti & Maklan [47] in Vermeir et al. [39].

#### 3.3.5. Construct Interference: Systematic Patterns

The literature suggests four systematic interference patterns:

Low institutional trust + High skepticism → Rejection: when consumers distrust certifiers and believe in generalized greenwashing, they opt out of sustainable categories [39].

High knowledge + Low defensive motivation → Systematic processing: informed consumers without pro-environmental self-concept investment evaluate claims analytically, reducing halo [19].

Multiple labels + Low knowledge → Confusion → Induced skepticism: information saturation among low-eco-literacy consumers increases perceived greenwashing, reducing predictive validity [46].

Halo effect + Affective heuristic → Confirmation bias: consumers attributing a positive halo to organic products selectively seek consistent information, reinforcing pre-existing beliefs, according to Weber [48] in Vermeir et al. [39].

#### 3.3.6. Methodological Implications and Research Agenda

**Figure 10:**
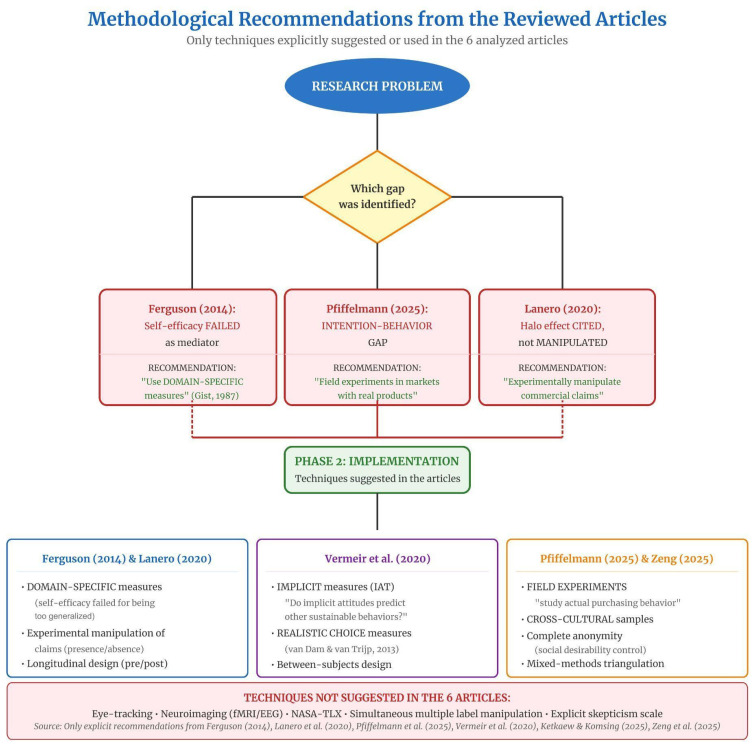
Methodological recommendations derived from the six reviewed studies, highlighting techniques explicitly proposed to address gaps in sustainable food behavior research. The figure translates identified limitations into actionable protocols: self-efficacy—where failure as a mediator (Ferguson, 2014 [17]) is attributed to overly general measures—calls for the adoption of domain-specific instruments (Gist [47]); the intention–behavior gap requires field experiments to assess actual purchasing behavior in realistic market contexts [20]; the halo effect demands experimental manipulation of commercial claims, addressing ambiguity and potential misinformation [19]; and implicit measures, together with realistic choice-based assessments, are needed to complement explicit attitudes, mitigating demand characteristics through between-subjects designs [39]. Techniques such as eye-tracking, neuroimaging (fMRI/EEG), NASA-TLX cognitive load assessments, simultaneous label manipulations, and explicit skepticism scales—although well established in the broader literature—were not proposed in the reviewed studies, thereby indicating clear opportunities for future research. Source: Authors’ elaboration based on [17,19,20,39,49,50].

This section analyzes cognitive biases in sustainable food decisions; Figure 10 and Table 17 summarize the resulting methodological recommendations.

### 3.4. External Validity and Behavioral Prediction

The preceding sections demonstrate that while psychometric instruments show strong internal validity, and DCEs capture stated preferences with increasing sophistication, both traditions operate within declarative paradigms. Their external validity—the degree to which findings predict real-world food purchases—remains largely untested. This section examines the four studies that bridge this gap by incorporating direct or observed behavioral measures, providing the critical benchmark against which declarative methods must be calibrated.

#### 3.4.1. Hierarchy of Behavioral Evidence

**Figure 11:**
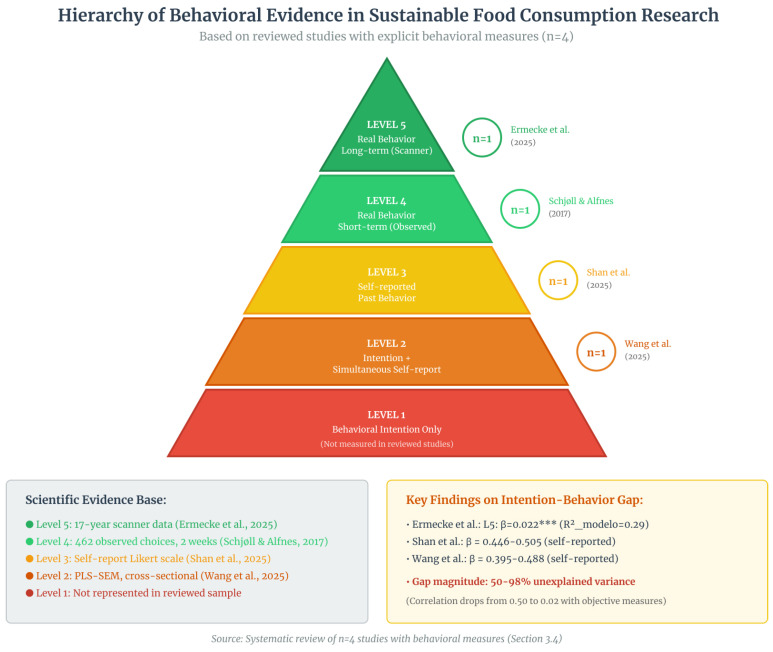
Hierarchy of behavioral evidence in the reviewed studies, illustrating the relative robustness of empirical approaches. The pyramid structure categorizes studies into five levels of behavioral evidence: Level 1—behavioral intention only (not represented in the reviewed sample); Level 2—simultaneous intention–behavior self-report; Level 3—retrospective self-reported behavior; Level 4—short-term observed behavior; and Level 5—longitudinal or objectively observed behavior. At the apex, Ermecke et al. [11] occupy the highest level with longitudinal scanner data spanning 17 years, representing 25% of the subsample. The intermediate levels include Schjøll and Alfnes [16] (25%), employing short-term observed behavior, while the lower levels concentrate 50% of studies—Shan et al. [12] and Wang et al. [23]—which rely on self-report without objective measurement. This distribution highlights methodological fragmentation: half of the studies operate with compromised predictive validity due to social desirability and recall biases, whereas only one-quarter achieve standards sufficient for robust behavioral inference. Note: Asterisks indicate statistical significance levels (*** *p* < 0.001). Source: Authors’ elaboration based on [11,12,13,16].

The four reviewed studies exhibit distinct levels of ecological validity, summarized in Figure 11. The figure organizes the studies into a hierarchical pyramid, from the lowest to the highest level of behavioral evidence.

#### 3.4.2. Types of Measures and Methodological Characteristics

Table 18 details the technical specifications of the behavioral measurements.

The distinction between the upper and lower levels of the pyramid (Figure 11) is reflected in the characteristics shown in Table 18: while Ermecke et al. [11] and Schjøll & Alfnes [16] capture behavior in real-purchase contexts, Shan et al. [12] and Wang et al. [13] rely on declarative reconstruction, subject to systematic distortions.

#### 3.4.3. Magnitude of the Intention–Behavior Gap

**Figure 12:**
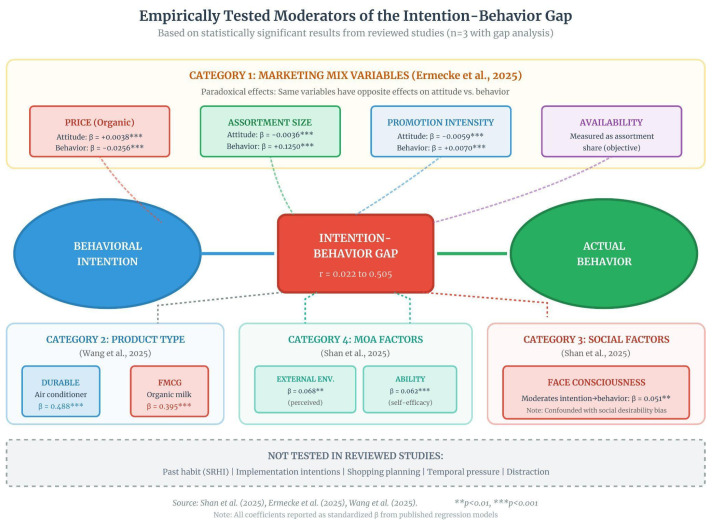
Empirically tested moderators of the intention–behavior gap in sustainable food behavior research. The figure synthesizes statistically significant effects across three studies, organizing moderators into four analytical categories: (1) marketing mix variables (price, assortment size, promotion intensity, and availability), which exhibit paradoxical effects across attitudes and behavior; (2) product type (durable vs. fast-moving consumer goods), influencing the strength of the intention–behavior relationship; (3) social factors, such as face consciousness, which moderate the translation of intention into action; and (4) MOA factors (motivation, opportunity, ability), capturing perceived environmental constraints and self-efficacy. Empirical evidence reveals substantial variation in effect sizes depending on measurement type. Objective scanner data (Ermecke et al. [11]) yield a statistically significant but practically negligible association (β = 0.022), whereas self-reported measures (Shan et al. [12]; Wang et al. [13]) produce moderate coefficients (β = 0.395–0.505). This divergence underscores the inflation of predictive validity in declarative measures relative to observed behavior. Note: Asterisks indicate statistical significance levels (** *p* < 0.01; *** *p* < 0.001). All reported coefficients correspond to standardized β estimates derived from regression-based models. Source: Authors’ elaboration based on [11,12,13].

Three studies allowed direct quantification of the relationship between psychological constructs and behavior. Figure 12 illustrates how this relationship varies according to the type of measurement.

Table 19 further quantifies these differences by comparing effect sizes across objective and self-reported behavioral data.

#### 3.4.4. Relationship Moderators

Figure 12 also maps the moderators tested in each study, organized into four categories. Visual analysis reveals three patterns:

First, marketing mix variables (Ermecke et al. [11]) display bidirectional arrows with distinct colors—red for negative effects on behavior, blue for positive effects on attitude. This “paradoxical effect” pattern indicates that the same factor (price, assortment, promotion) operates in opposite directions on psychological evaluations versus actual purchase decisions.

Second, MOA factors (Shan et al. [12]) are concentrated at the center of the figure, with green arrows indicating moderate positive effects. Emotional Information (β = 0.088) shows a larger magnitude than Rational Information (β = −0.006, not significant), suggesting affective predominance over cognitive processing in mediating the relationship.

Third, product category (Wang et al. [13]) appears isolated in the lower panel, with a color distinction between durables (blue, β = 0.488) and FMCG (red, β = 0.395). The proximity of these values in the figure—compared to the distance between Ermecke et al. [11] and the others—suggests that within self-reported data, variations are smaller than those between self-report and objective behavior.

#### 3.4.5. Methodological Triangulation

**Figure 13:**
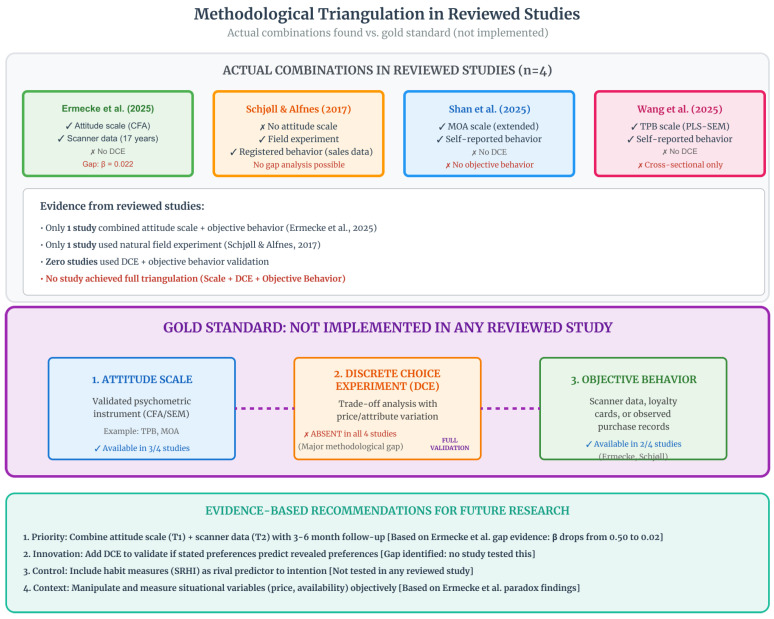
Methodological triangulation in sustainable food behavior research. Existing combinations versus the missing gold standard. The lower section highlights the gold-standard combination—scale + discrete choice experiment (DCE) + actual behavior—marked with a golden star and explicitly labeled “NOT IMPLEMENTED.” This gap is epistemological: no study sits at the intersection of the three methods, leaving the literature without evidence on the predictive validity of DCEs, the incremental variance explained by psychometric scales, or the combined limitations of declarative methods. The figure emphasizes “methodological silos”: studies including observed behavior omitted DCEs; studies using DCEs (none in the sample) lacked behavioral validation; studies relying on scales dominate but without external validation. Source: The authors, based on [11,12,13,16].

Figure 13 presents the matrix of methodological combinations. The upper section shows the four studies in their actual configurations: three of them (75%) share the absence of DCE, and two (50%) share the absence of objective behavior measurement.

Table 20 details these combinations, specifying the methodological gap in each study. Ermecke et al. [11], despite achieving the highest level of behavioral validity, omitted the DCE, preventing assessment of whether preferences elicited in discrete choice experiments predict scanner data behavior. Schjøll & Alfnes [16], by excluding intention scales, made analysis of the intention–behavior gap impossible. Shan et al. [12] and Wang et al. [13], focused on self-report, lack both objective behavior and DCE, limiting themselves to face validity.

#### 3.4.6. Methodological Integration and Predictive Architecture

**Figure 14:**
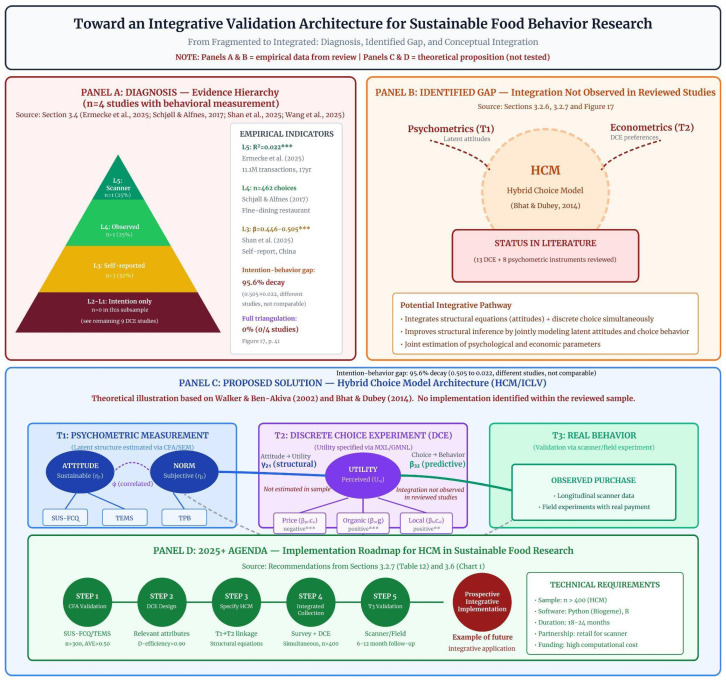
Scientific validation architecture for sustainable food behavior research. Panel (**A**) Illustrates the concentration of evidence within declarative paradigms, highlighting the predominance of self-report measures. Panel (**B**) Highlights the absence of studies that combine validated psychometric instruments, discrete choice experiments (DCEs), and objective behavioral data within a single structural design. Panel (**C**) Proposes a structural articulation of these components to enable integrated analysis. Panel (**D**) Outlines a progressive roadmap for methodological integration, guiding future research toward higher validity and comprehensive behavioral inference. Source: Authors’ elaboration based on [11,12,13,17,39]. Notes: ** *p* < 0.05; *** *p* < 0.01.

To synthesize the structural fragmentation identified across the reviewed studies, Figure 14 presents a scientific validation architecture for sustainable food behavior research.

This section synthesizes the implications of the methodological fragmentation identified in the reviewed literature and proposes a conceptual architecture for cumulative predictive research in sustainable food behavior.

A central finding emerging from the systematic comparison of methodological approaches is the structural disarticulation between psychometric measurement, experimental choice modeling, and behavioral observation. While each of these traditions has developed sophisticated analytical tools, they have evolved largely in isolation rather than through coordinated methodological integration.

Psychometric instruments grounded in behavioral theories have achieved high levels of structural validity and measurement invariance. Instruments such as the Food Choice Questionnaire and its derivatives provide robust measurement of motivational constructs and sustainability-related attitudes. At the same time, econometric approaches based on Random Utility Theory have produced increasingly refined models capable of estimating heterogeneous consumer preferences through discrete choice experiments and advanced logit specifications. Behavioral studies using scanner data or field experiments have, in turn, generated ecologically valid evidence about real purchasing decisions.

Despite these advances, the three methodological traditions rarely converge within the same empirical design. As a result, sustainable food behavior tends to be modeled in analytical fragments. Attitudes are measured without structural embedding in econometric decision models; marginal utilities are estimated without incorporating validated latent constructs; and observed behavior is analyzed without systematic reference to psychological mechanisms.

This fragmentation generates an inverted hierarchy of evidence in which the largest share of empirical production remains concentrated in self-report studies measuring attitudes and intentions in hypothetical contexts. Such studies frequently report moderate and statistically significant associations between attitudes and behavioral intentions. However, when behavior is measured using objective scanner data across extended time horizons, the magnitude of this relationship decreases substantially, revealing a structural misalignment between declarative models and real-world decision environments.

Declarative measures are particularly vulnerable to social desirability bias, hypothetical bias, and the absence of real economic consequences. Without calibration against observed behavior, estimates of sustainable consumption may therefore be systematically inflated. If the goal of consumer research is reliable prediction under real constraints, near-exclusive reliance on declarative measures represents not only a methodological limitation but also an epistemological barrier to cumulative knowledge.

Psychological mechanisms further reinforce this fragmentation. Evidence indicates that trust, skepticism, and heuristic processing systematically mediate the translation of attitudes into behavior. Heuristic processes such as halo effects, affective heuristics, and default choices influence decisions independently of explicit sustainability attitudes. Consumers may infer health or taste attributes from sustainability labels, respond emotionally to environmental framing, or follow default options without conscious evaluation.

Trust in sustainability claims also varies across domains. Consumers often differentiate between environmental, ethical, and health-related claims, and skepticism toward greenwashing can attenuate or completely neutralize the effect of positive environmental attitudes. Yet skepticism remains insufficiently operationalized as a latent construct in most structural models, limiting the explanatory power of existing behavioral frameworks.

To articulate these elements within a unified analytical structure, Table 21 organizes sustainable food behavior research into a multilevel functional architecture. The framework integrates seven analytical layers ranging from macro-contextual constraints to behavioral validation procedures.

Within this framework, sustainable choice can be formally represented as a probabilistic decision process derived from Random Utility Theory:

$$P_{ij}=\frac{e^{U_{ij}}}{\sum_{k}e^{U_{ik}}}$$

Utility is conceptualized as a joint function of observable attributes, latent motivations, heuristic moderators, and contextual constraints.

**Figure 15:**
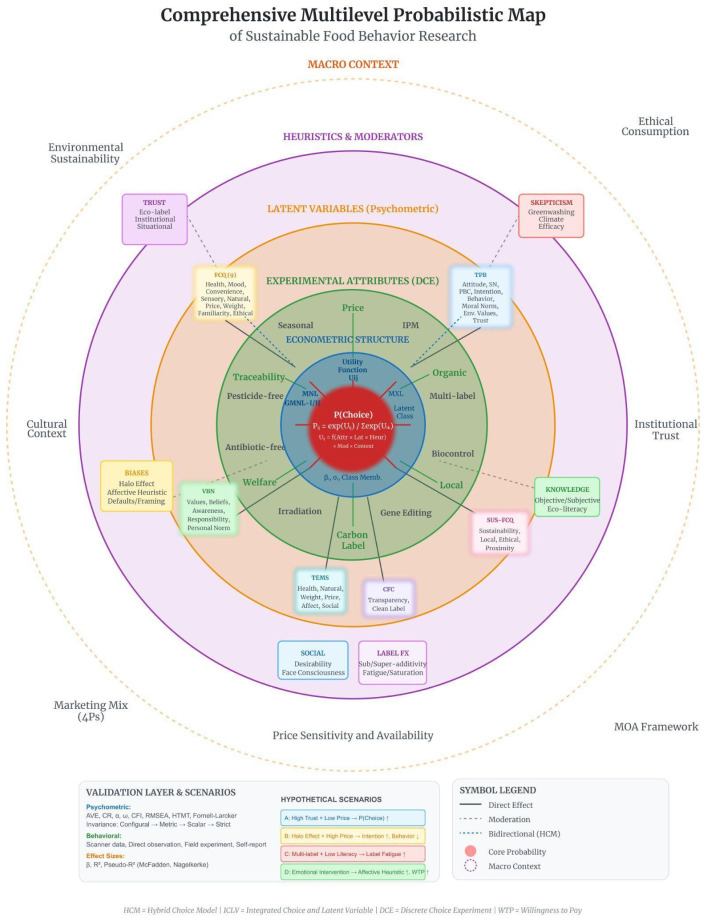
Comprehensive multilevel probabilistic ecosystem of sustainable food behavior. The figure synthesizes evidence into nested analytical layers: Core—Probabilistic Choice: Based on Random Utility Theory; observable product attributes (price, certifications, local origin, carbon labels, animal welfare) drive marginal utilities and willingness to pay. Experimental & Econometric Layer: Design space of discrete choice experiments, with heterogeneity captured via Mixed Logit, GMNL, latent class, and Integrated Choice and Latent Variable (ICLV) models. Latent Psychological Variables: Motivations (TEMS), sustainability attitudes (SUS-FCQ), values and norms (VBN), and temporal orientation (CFC) influence utility structurally, rather than directly determining behavior. Heuristics and Cognitive Moderators: Trust, skepticism, halo effects, affective heuristics, and social desirability modify the translation of attitudes into action, explaining intention–behavior gaps. Macro-Contextual Environment: Cultural norms, institutional trust, marketing exposure, price sensitivity, and product availability shape consumer decision spaces and the effectiveness of psychological motivations. Together, the figure emphasizes that sustainable food behavior emerges from the interaction of psychological, econometric, and contextual factors. It illustrates the need for integrated Hybrid Choice Models that combine psychometric rigor, experimental design, and behavioral validation, bridging methodological fragmentation toward cumulative predictive understanding. Source: The authors, based on [1,2,3,6,7,8,9,10,11,15,16,17,18,19,20,21,24,25,39,51,52].

Figure 15 synthesizes this architecture as a comprehensive multilevel probabilistic ecosystem of sustainable food behavior.

Hybrid Choice Models (HCM/ICLV) provide the econometric structure capable of estimating this integrated system. These models combine discrete choice experiments with latent variable models, allowing psychological constructs to influence utility functions indirectly through structural relationships. In doing so, they offer a formal framework through which motivational constructs measured through psychometric instruments can be incorporated into econometric choice models derived from Random Utility Theory. However, none of the studies included in the present review implemented a full empirical configuration combining validated psychometric scales, discrete choice experiments, and objective behavioral validation within a single research design.

The absence of such integrated studies can be explained by several practical constraints that limit the feasibility of large-scale methodological integration. Implementing hybrid modeling frameworks requires strong theoretical specification, large sample sizes, advanced econometric expertise, and access to multiple forms of data collected through different methodological procedures. In particular, the acquisition of objective behavioral data represents a significant barrier. Retailers are often reluctant to share scanner data due to commercial confidentiality, while field experiments capable of generating behavioral observations require substantial financial and logistical resources. At the same time, editorial dynamics within academic publishing frequently favor shorter empirical studies with clearly delimited methods rather than large integrated research designs combining several methodological traditions.

For these reasons, methodological integration should be understood as a progressive research agenda rather than an immediate empirical standard. Psychometric rigor remains indispensable for identifying latent motivational structures underlying sustainable consumption decisions. Econometric modeling remains necessary for estimating decision probabilities and preference heterogeneity within market environments. Behavioral validation, in turn, remains the ecological benchmark through which the predictive capacity of theoretical models can ultimately be evaluated. The challenge facing the field is therefore not the replacement of existing methodological paradigms but their articulation within a coherent analytical architecture.

A second methodological issue concerns the design and optimization of survey instruments used to measure psychological constructs. The analysis of the instruments mapped in the present review reveals a recurrent tension between psychometric robustness and data collection feasibility. Instruments developed with strong theoretical foundations often contain a large number of items in order to ensure adequate coverage of conceptual dimensions. However, excessively long questionnaires increase respondent fatigue, dropout rates, and satisficing behavior, thereby compromising the quality of the collected data. Notably, none of the studies included in the sample reported information regarding questionnaire completion time, dropout rates, or indicators of cognitive load during survey administration. The absence of such information represents an additional limitation in terms of ecological validity and practical applicability.

Based on the methodological analysis conducted in this review and on the literature concerning survey design and cognitive load, several parameters can be proposed to guide the optimization of future instruments. Survey completion time should ideally remain within a range of 5 to 10 minutes, with 15 minutes representing an upper practical limit in most online data collection contexts. The total number of items should preferably remain between 10 and 20, with a maximum threshold of approximately 30 items in cases where additional dimensions must be measured. Within each construct, a minimum of three to four items is necessary to ensure measurement stability, although five to eight items per dimension generally provide a more robust representation of latent variables. In addition, acceptable survey dropout rates should remain below 20 percent for questionnaires designed to be completed within a 5-to-10-minute timeframe.

Achieving these optimization goals without compromising psychometric validity requires methodological procedures capable of identifying the most informative items within an instrument. Iterative applications of exploratory and confirmatory factor analysis can be used to eliminate items presenting high cross-loadings or insufficient factor loadings, thereby refining the dimensional structure of the scale. Complementarily, Item Response Theory (IRT) models allow researchers to estimate the informational value of individual items across different levels of the latent trait, enabling the selection of items that provide the highest psychometric information relative to response time. In addition to statistical procedures, qualitative pretesting methods also play an essential role in improving instrument quality. Cognitive interviews and think-aloud protocols allow researchers to identify comprehension difficulties, ambiguous wording, or excessive cognitive effort required during survey completion before large-scale data collection begins.

Taken together, these procedures suggest a structured process for instrument development that begins with conceptual definition and expert validation of an initial item pool, followed by cognitive pretesting with small samples in order to identify comprehension problems. Subsequent stages involve statistical item selection based on factor analytic and IRT criteria, with the goal of producing a reduced instrument capable of being completed within a limited timeframe while preserving adequate psychometric properties. The final stage consists of cross-validation in independent samples through confirmatory factor analysis and the empirical measurement of completion time and dropout rates.

Finally, several limitations of the present review must be acknowledged. The corpus analyzed is characterized by considerable geographical and temporal heterogeneity, which restricts direct comparability across contexts and consumption environments. In addition, the search cutoff date of February 2025 temporally bounds the conclusions in a field that is evolving rapidly in response to new methodological and technological developments. The inclusion criteria adopted in the review also introduced certain restrictions. In particular, the requirement that psychometric studies include confirmatory factor analysis and that discrete choice experiments provide explicit econometric model specification led to the exclusion of some exploratory studies and alternative measurement approaches that may nonetheless offer relevant methodological contributions.

Future research should therefore expand search strategies and inclusion criteria in order to capture a broader diversity of methodological approaches. More fundamentally, however, progress in the field will depend less on multiplying isolated methodological applications and more on constructing coordinated research designs capable of integrating psychometric measurement, experimental modeling, and behavioral validation within a cumulative predictive framework. Only through such integration will it be possible to reduce the fragmentation that currently characterizes sustainable food behavior research and to advance toward more reliable and ecologically grounded behavioral prediction.

## 4. Conclusions

This review demonstrates that the primary limitation in sustainable food behavior research lies not in the absence of methodological sophistication, but in the persistent fragmentation between psychometric measurement, econometric modeling, and behavioral validation. While each tradition has advanced independently, their lack of integration results in partial analytical representations of consumer behavior, in which attitudes, preferences, and observed actions are rarely examined within a unified framework. The evidence reveals a systematic gap: no study currently combines validated psychometric instruments, discrete choice experiments, and objective behavioral data in a single design, limiting the field’s capacity to assess predictive validity and to develop cumulative explanations of sustainable purchasing behavior. Addressing this limitation requires a progressive integration of methodological approaches, aligning latent psychological constructs, experimental choice modeling, and real-world behavioral data. Such integration is essential to move beyond declarative inference and toward robust, ecologically valid prediction of consumer behavior under real-world constraints.

These findings position methodological integration not merely as a technical improvement, but as a necessary condition for advancing cumulative, policy-relevant knowledge in sustainable food behavior.

## Figures and Tables

**Table 1:** Evolution of Models in Food Choice Research.

Year	Instrument	Theoretical & Structural Basis	Conceptual Contribution
1974	Multinomial Logit (MNL)	Random Utility Theory; Uij = Vij + εij; Independence of Irrelevant Alternatives (IIA) property	Formal probabilistic foundation of discrete choice modeling
1991	Theory of Planned Behavior (TPB)	Social Cognitive framework; intention model; belief-based constructs	Integration of cognitive determinants into behavior prediction
1995	Food Choice Questionnaire (FCQ)	Health Psychology; Exploratory Factor Analysis (EFA) and Confirmatory Factor Analysis (CFA)-derived 9-factor latent structure	Operationalization of multidimensional food motives
2003	Mixed Logit (MXL)	Random coefficients model; simulated maximum likelihood	Incorporates unobserved preference heterogeneity
2010	Generalized Multinomial Logit (GMNL)	Random coefficients + scale heterogeneity parameter	Separates taste and scale heterogeneity
2017	The Eating Motivation Survey-Brazil (TEMS-BR)	Motivational Psychology; validated multi-factor scale	Cross-cultural adaptation and contextual refinement

**Table 2:** Defining Characteristics of the Generations of Instruments.

Generation	Instrument	Innovation	Limitation
1st (1990–2005)	Food Choice Questionnaire (FCQ)	Multidimensional food motivation model (9 factors)	Sustainability subsumed under health
2nd (2005–2015)	Theory of Planned Behavior (TPB) extensions; Value–Belief–Norm (VBN) framework	Incorporation of moral norm and pro-environmental constructs	Generic sustainability focus (not food-specific)
3rd (2015–2020)	Product-specific models; Veg & Private Label studies	Segmentation and closer approximation to actual choice	Context-dependent, low replication
4th (present)	Sustainable Food Choice Questionnaire (SUS-FCQ); Clean Food Consumerism (CFC); The Eating Motivation Survey–Brazil (TEMS-BR)	Integrated sustainability constructs; multigroup invariance; second-order models	Persistence of intention–behavior gap

**Table 3:** Instrument Operationalization by Analytical Level.

Level	Core Constructs	Main Instruments	Example of Application
Macro	Environmental sustainability, ethics, cultural context	Sustainable Food Choice Questionnaire (SUS-FCQ) (Local and Seasonal), Theory of Planned Behavior–Moral Norm (TPB-NM)	Compare priorities between developed and developing countries
Meso	Attitude, norms, Perceived Behavioral Control (PBC), intention	Classical and extended Theory of Planned Behavior (TPB), Image Theory	Predict adoption of sustainable innovations (plant-based eggs, alternative proteins)
Micro	Immediate motivations (health, price, naturalness)	Food Choice Questionnaire (FCQ), the Eating Motivation Survey (TEMS), Clean Food Consumerism (CFC)	Segment consumers by motivational profile

**Table 4:** First-Generation Instruments: Simple Factorial Structure.

Study	Model/ Structure (C; I)	Psychometrics	Context & Limitations
[5]	Theory of Planned Behavior (TPB) + Value–Attitude–Behavior (VAB)	Comparative Fit Index (CFI) = 0.933; Root Mean Square Error of Approximation (RMSEA) = 0.088; Average Variance Extracted (AVE) > 0.75; Cronbach’s alpha (α) > 0.83	Taiwan, plant-based eggs; negative Perceived Behavioral Control (PBC) (standardized regression coefficient (β) = −0.525; no behavior measured
[4]	Extended Reasoned Personal Involvement (ERPI)/Theory of Planned Behavior (TPB)	Comparative Fit Index (CFI) = 0.958; Root Mean Square Error of Approximation (RMSEA) = 0.055; Cronbach’s alpha (α) > 0.90	4 Latin American countries; young sample; no behavior tested
[26]	Veg Private Labels	Comparative Fit Index (CFI) = 0.959; coefficient of determination (R^2^) = 64.4% vs. 38.6%; Cronbach’s alpha (α) = 0.970	Italy; 88% women; redundancy risk

Interpretive Synthesis: These models explain intentions rather than actual behaviors. The variation in predictive power (R^2^) across segments suggests that they are more effective for consumers already committed to the sustainable category.

**Table 5:** Level 2 Instruments.

Instrument/ Study	Structure	Invariance	Psychometrics	Restriction
Sustainable Food Choice Questionnaire (SUS-FCQ) [7]	2 factors; 9 items	Configural, Metric, Partial Scalar (5 European countries)	Comparative Fit Index (CFI) > 0.95; Root Mean Square Error of Approximation (RMSEA) < 0.08; Average Variance Extracted (AVE) > 0.50; Cronbach’s alpha (α) = 0.89–0.93	Caution in mean comparisons; behavior not tested
Clean Food Consumerism (CFC) [28]	5 factors → second-order; 18 items	Configural, Metric, Partial Scalar (Israel vs. UK)	CFI = 0.97; RMSEA = 0.033; AVE = 0.55–0.76; coefficient of determination (R^2^) = 79%	Sustainability implicit (via transparency), not explicit
The Eating Motivation Survey-Brazil (TEMS-BR) [6]	8 factors; 24 items	Full strict invariance (sex, age, BMI)	CFI = 0.96; RMSEA = 0.06; AVE = 0.51–0.75; McDonald’s omega (ω) = 0.68–0.87	Reduction (45→24 items); Acre-only sample

Interpretive Synthesis: The Eating Motivation Survey-Brazil (TEMS-BR) establishes the gold standard for national validation, as it is the only instrument demonstrating full strict invariance, enabling reliable comparisons across demographic groups. The Sustainable Food Choice Questionnaire (SUS-FCQ) is the only sustainability-specific food choice instrument with international validation. The Clean Food Consumerism (CFC) introduces second-order modeling, allowing “clean food consumerism” to be operationalized as a hierarchical construct.

**Table 6:** Level 3 Instruments (Jayawardhena et al., 2016 [25]; Naspetti et al., 2021 [3]).

Dimension	[25] Jayawardhena et al. (2016)	[3] Naspetti et al. (2021)
Instrument	Image Theory Scale	Theory of Planned Behavior (TPB) + Moral Norm (Dairy)
Structure	9 factors → 3 s-order factors, 22 items	4 factors, 12 items
Actual Behavior	Yes: Retrospective purchase (2 weeks: Fair Trade, organic, non-GMO)	No (intention only)

Interpretive Synthesis: The Image Theory Scale is the only instrument in the full sample that incorporates actual behavioral measures, offering a theoretical advantage by distinguishing between value images, trajectory images, and strategic images. The study by Naspetti et al. demonstrates theoretical robustness by maintaining a stable structure across three distinct practices (agroforestry, prolonged breastfeeding, and alternative protein adoption).

**Table 7:** Overlap Matrix of Core Constructs (Estimated Theoretical Correlations).

Construct	Sustainable Food Choice Questionnaire (SUS-FCQ) (Sustainable)	Clean Food Consumerism (CFC) (Transparency)	The Eating Motivation Survey (TEMS) (Natural Concerns)	Theory of Planned Behavior–Moral Norm (TPB-NM)	Image Theory (Values)
Environmental Concern	0.85	0.45	0.72	0.68	0.55
Animal Welfare	0.78	0.30	0.65	0.70	0.48
Personal Health	0.42	0.88	0.58	0.35	0.62
Transparency/Clean Label	0.65	0.92	0.48	0.52	0.58
Ethical Consumption	0.90	0.55	0.60	0.85	0.75
Proximity/Local	0.88	0.35	0.55	0.45	0.40

Note: Values > 0.80 in bold indicate high overlap. Source: estimates based on reported factor loadings and Average Variance Extracted (AVE). Critical insight: The estimated correlation of 0.90 between Sustainability (SUS-FCQ) and Consumption Ethics (Theory of Planned Behavior–Moral Norm (TPB-NM)) indicates that consumers do not clearly distinguish environmental concerns from general moral norms, suggesting the need for integrated operationalization in future instruments. In contrast, Transparency/Clean Label (CFC) correlates more strongly with Personal Health (0.88) than with Environmental Concern (0.45), revealing that “clean food” is conceptually closer to health motivations than to environmental sustainability per se.

**Table 8:** Recommendation Matrix by Research Scenario.

Research Scenario	Recommended	Justification	Required Adaptation
Actual purchase behavior	Image Theory Scale	The only instrument with direct behavioral validation; coefficient of determination (R^2^) = 20% for Fair Trade and organic products	Replication in the Brazilian context; probabilistic sample
International comparison	Sustainable Food Choice Questionnaire (SUS-FCQ)	Validated in 5 European countries + Turkey; metric equivalence established	Adaptation for developing countries; full scalar invariance
National comparison (Brazil)	The Eating Motivation Survey-Brazil (TEMS-BR)	The only instrument with full strict invariance (4 levels) by sex, age, BMI	Expansion to other regions; behavioral validation
Specific sustainable innovations	Theory of Planned Behavior (TPB) + Value-Attitude − Behavior (VAB) model (plant-based eggs) or Veg Private Labels	Flexibility for adaptation; segmentation of occasional vs. regular consumers	Model respecification; reduction of gender bias
Clean label/transparency	Clean Food Consumerism (CFC)	Robust second-order structure; excellent Root Mean Square Error of Approximation (RMSEA) (0.033)	Translation into Portuguese; test of actual behavior
Comparative public policies	Sustainable Food Choice Questionnaire (SUS-FCQ) or Theory of Planned Behavior (TPB) + Moral Norm (TPB-NM)	SUS-FCQ: food-specific focus; TPB-NM: strong explanatory power (coefficient of determination (R^2^) = 57–68%)	Integration into a hybrid model

**Table 9:** Methodological Synthesis of Scale, Instrument, and Model Adoption.

Scale (Point Likert)	Instrument (ID)	Model/Core Structure	When and How to Use
5	The Eating Motivation Survey-Brazil (TEMS-BR) (Instrument Identifier (ID) 13)	Level 2: Multidimensional with Measurement Invariance	NATIONAL COMPARISON (Brazil): Ideal for comparing demographic groups (gender, age, BMI), as it demonstrates full strict invariance. Recommended when reducing cognitive burden is a priority.
5	Clean Food Consumerism (CFC) (Instrument Identifier (ID) 1)	Level 2: Second-Order Structure	TRANSPARENCY/HEALTH FOCUS: More strongly associated with Personal Health than Environmental Sustainability. Recommended for studies on “clean food” and transparency-related motivations.
7	Image Theory Scale (Instrument Identifier (ID) 6)	Level 3: Hierarchical/Actual Behavior	HIGHEST PRIORITY (Gold Standard): Measures actual purchasing behavior (e.g., Fair Trade, organic products), mitigating intention bias. Ideal for studies requiring high ecological predictive validity.
7	Extended Theory of Planned Behavior (TPB) with Moral Norm (Instrument Identifier (ID) 14)	Level 3: Extended TPB/Moral Norm	EXPLICIT SUSTAINABILITY/PUBLIC POLICY: Strong explanatory power for pro-environmental and moral motivations. Seven-point scales increase variance in regression and Structural Equation Modeling (SEM) models.
7	Theory of Planned Behavior (TPB) + Value-Attitude-Behavior (VAB) Model (Instrument Identifier (ID) 3)	Level 1: Simple Factorial Structure/TPB	SPECIFIC INNOVATIONS: Suitable for predicting adoption intentions of specific innovations (e.g., plant-based eggs). Should be adapted to the product under study.
7	Composite Model (Veg Private Labels) (Instrument Identifier (ID) 2)	Level 1: Simple Factorial Structure/Rational Action (RA)	CONSUMER SEGMENTATION: Useful for distinguishing regular vs. occasional buyers and comparing motivational differences and predictive power (coefficient of determination (R^2^)) across segments.
Not Specified	Sustainable Food Choice Questionnaire (SUS-FCQ)	Level 2: Multidimensional with Measurement Invariance	INTERNATIONAL COMPARISON: European validation. Explicit focus on Sustainability and Local & Seasonal dimensions.

**Table 10:** Status of Psychometric Properties in the Mapped Sample.

Dimension	Status	Example	Priority Gap	Recommendation
Structural validity	Good (7/8 with Confirmatory Factor Analysis (CFA))	The Eating Motivation Survey-Brazil (TEMS-BR), Clean Food Consumerism (CFC), Sustainable Food Choice Questionnaire (SUS-FCQ)	Replication in probabilistic samples	Conduct Exploratory Factor Analysis (EFA) + Confirmatory Factor Analysis (CFA) in independent samples
Convergent validity	Adequate (Average Variance Extracted (AVE) > 0.50 in 6/8)	All Level 2 and 3 studies	Exclusive use of Fornell–Larcker criterion	Adopt Heterotrait–Monotrait Ratio (HTMT) as a complementary criterion
Discriminant validity	Partial (5/8 tested)	Veg Private Labels, TEMS-BR	Tests absent in 37.5%	Make Fornell–Larcker criterion + Heterotrait–Monotrait Ratio (HTMT) mandatory
Predictive validity	Critical (1/8 with actual behavior)	Image Theory (only one)	Intention–behavior gap in 87.5%	Prioritize purchase diaries, loyalty card data
Measurement invariance	Partial (5/8 tested; 1 full)	The Eating Motivation Survey-Brazil (TEMS-BR) (full)	Strict invariance absent in 7/8	Test strict invariance as standard practice
Temporal stability	Absent (1/8 with test–retest)	Sustainable Food Choice Questionnaire (SUS-FCQ) Turkey (Intraclass Correlation Coefficient (ICC) = 0.689)	Temporal consistency unknown	Include 2–4-week test–retest
Ecological validity	Absent (1/8 in natural setting)	—	Controlled setting vs. real decision	Conduct ethnographic studies in supermarkets

**Table 11:** Decision Matrix for Selecting Econometric Models in Sustainable Food Research.

Research Question	Recommended Model	Technical Specification	Minimum n	Reference
“What is the average population WTP?” (limited resources)	Conditional Logit (Multinomial Logit (MNL))	Homogeneous preferences; point estimate	>100	[8]
“Are there distinct consumer segments?” (targeted policies)	Mixed Logit (MXL)	Random parameters with normal distribution; captures distributional heterogeneity	>200	[10]
“Are attributes correlated?” (e.g., organic × local)	MXL with correlation	Unrestricted covariance matrix (Σ)	>300	[10]
“Do respondents have different certainty levels?” (rigorous validation)	Generalized Multinomial Logit (GMNL-II)	Certainty scale as individual-specific scale parameter (σ_i_)	>500	[9]
“How do attitudes influence choices?” (behavioral theory)	Hybrid Choice Model/Integrated Choice and Latent Variable (HCM/ICLV)	Structural equations (latent attitudes) + simultaneous choice equations	>400	Not implemented in the reviewed studies
“How do interventions change preferences?” (pre/post experiment)	MXL with interactions	Within-subjects design; attribute × experimental condition interaction	>400	[15]

Note: Computational cost increases exponentially: MNL (seconds) → MXL (minutes) → MXL+ (hours) → GMNL (hours–days) → HCM (days).

**Table 12:** Willingness-to-Pay (WTP) Hierarchy by Categories of Sustainable Attributes in Discrete Choice Experiments (DCEs).

Attribute Category	Reported Mean WTP	Range	Reference Studies	Identified Mechanism
Organic certification	€1.36–$5.76	High variability	[8,10,15]	Familiarity effect: WTP 863% higher among consumers familiar with the label
Animal welfare	€2.44	[€1.09; €7.26]	[8]	Extreme heterogeneity by demographic segment (mature women: €7.26 vs. millennials: €1.09)
Local/national origin	€2.01–€11.40	[€2.01; €11.40]	[8,32]	Food “home bias”; cultural protectionism
Integrated Pest Management (IPM)	$1.10–$3.40	[$1.10; $3.40]	[9]	Technology aversion: biocontrol > gene editing > irradiation
Carbon labels	−$0.44 to +$1.12	Conditional effect	[15]: −¥0.44 (baseline); +¥0.91 (post-intervention)	Informational priming effect: climate change information increases WTP by 109%
Multi-level eco-labels	€0.33–€1.37	[€0.33; €1.37]	[14]: Planet Score B (€1.37) vs. D (€0.33)	Environmental literacy required to interpret graded formats

Note: Converted from original currencies using 2025 average exchange rates (EUR/USD: 0.92; EUR/CNY: 0.13). Original studies reported values between 2018 and 2025; no inflation adjustment was applied as the range already captures temporal variation. Emerging Pattern: Direct human health attributes (pesticide and antibiotic residues) consistently outweigh diffuse environmental impact attributes (carbon footprint, biodiversity), confirming the “proximity hierarchy” in pro-environmental behavior [37].

**Table 13:** Hierarchy of Rigor in Mitigating Hypothetical Bias in Discrete Choice Experiments (DCEs).

Level	Strategy	Frequency (*n* = 13)	Implementation	Estimated Effectiveness
**Level 1**—Experimental Design (Ex ante)	Cheap talk script	23.1% (3/13)	[9]	40% bias reduction [38]
	Realistic scenario contextualization	61.5% (8/13)	Detailed product description	Low (no measurable isolated effect)
	Explicit opt-out	84.6% (11/13)	Standard in most studies	Moderate (increases decision realism)
**Level 2**—Instrumentation (Per-task)	Certainty scale (0–10)	7.7% (1/13)	[9]	High (when incorporated into the GMNL model)
	Attention/attribute checks	15.4% (2/13)	[15]	Moderate (filters inattentive respondents)
	Response time monitoring	7.7% (1/13)	[38]	Moderate (processing indicator)
**Level 3**—Modeling (Ex post)	Incorporation of certainty scale into GMNL	7.7% (1/13)	[9]	High (controls for scale heterogeneity)
	Exclusion of low-certainty respondents	0% (0/13)	—	Potentially high (reduces noise)
**Level 4**—External Validation (Gold Standard)	Comparison with scanner data	0% (0/13)	—	Absolute benchmark
	Longitudinal behavioral follow-up	0% (0/13)	—	Absolute benchmark
	Field experiment with real payment	0% (0/13)	—	Absolute benchmark

**Table 14:** Levels of Integration between Psychometric Constructs and Discrete Choice Experiment (DCE) Models.

Dimension	Level 1: Descriptive Use (61.5%)	Level 2: Correlational Use (23.1%)	Level 3: Structural Use (0%)
Definition	Psychometric scales used for sample characterization; a posteriori correlations with utility parameters	Psychological variables included as covariates in the utility function or class membership equation	Simultaneous modeling of structural equations (latent attitudes) and choice equations via Hybrid Choice Model/Integrated Choice and Latent Variable (HCM/ICLV) models
Frequency	8/13	3/13	0/13
Studies	Kolber & Meixner: correlation between New Ecological Paradigm (NEP) and eco-label importance (r = 0.346) [14]	Gallardo et al. [9]: 12 purchase motives as class covariates; Liu et al. [15]: experimental group as moderator	—
Limitation	Does not model causal mechanisms; loss of explanatory power	Indirect integration; does not capture latent attitudinal structure	CRITICAL GAP: None of the 13 DCE studies reviewed tested how attitudes shape preferences within a causal structure, highlighting the need for integrated Hybrid Choice Models

**Table 15:** Methodological Evolution in DCEs for Sustainable Foods.

Dimension	Status 2018–2020	Status 2022–2025	Recommendation for 2025+
Econometric modeling	Predominance of Basic Conditional Logit (CL)/Multinomial Logit (MNL) models	Widespread adoption of Mixed Logit (MXL); emergence of Generalized Multinomial Logit (GMNL) and Latent Class	Prioritize Generalized Multinomial Logit II (GMNL-II) or Hybrid Choice Model (HCM) to capture actionable heterogeneity
Experimental design	Predominantly fractional orthogonal designs	D-efficient designs in 30.8%; efficiency metrics reported	Mandatory reporting of D-efficiency; use of adaptive designs
Bias mitigation	Cheap talk in <25% of studies	Certainty scale incorporated into the model (9)	Adoption of “lab-in-the-field” protocols with real monetary payment
External validation	Absent	Still absent in 100% of studies	CRITICAL PRIORITY: Retail partnerships for real-market acceptance testing

Central Implication: Methodological advances from Eldesouky [8] to Gallardo [9] show that sophisticated models reveal preference structures invisible to basic approaches. However, the lack of external validation prevents translating these insights into reliable market projections.

**Table 16:** Construct Summary: Measurement Status, Key Evidence, and Critical Gaps.

Construct	Status	Key Evidence	Critical Gap
Trust (context-specific)	Measured	Cronbach’s alpha (α) = 0.91; Average Variance Extracted (AVE) = 0.66 (Ferguson, 2014); standardized regression coefficient (β) = 0.41 *** [21]	Does not distinguish institutional vs. situational vs. behavioral trust
Skepticism	Not measured	Discussed as a barrier [17]; qualitatively identified [18]; moderator in external studies [42]	No latent variable in reviewed models; skepticism × trust interaction not tested
Halo effect	Not tested	Cited as a conceptual gap [17]; credibility × judgment correlation (r) = 0.58 [19]	No experimental manipulation of labels in the reviewed studies
Defaults/heuristics	Measured	Standardized regression coefficient (β) = 0.84 ***—strongest effect on willingness to pay (WTP) [17]; operationalized via opt-in/opt-out scenarios	Evidence limited to a single study; not replicated in other contexts

Note: Asterisks indicate statistical significance levels (*** *p* < 0.001). Reported β coefficients are standardized.

**Table 17:** Methodological recommendations explicitly suggested in the reviewed articles.

Problem	Recommendation	Source
Generalized self-efficacy fails as a mediator	Use measures SPECIFIC to the sustainable purchasing domain	Zeng et al. [21], citing Gist [22]
Halo effect not isolated	Experimentally manipulate commercial claims (presence/absence)	Lanero et al. [23]
Intention–behavior gap	Conduct field experiments in markets with real products	Pfiffelmann et al. [24]
Reliance on self-report	Use IMPLICIT measures (IAT) + realistic choice measures	Vermeir et al. [25]
Social desirability bias	Full anonymity + between-subjects design	Vermeir et al. [25]; Ketkaew & Komsing [26]
Limited generalizability	Cross-cultural samples; replication in other countries	Stern et al. [24]; Martinelli et al. [27]

**Table 18:** Characteristics of Behavioral Measures in the Four Studies.

Study	Type of Measure	Specification	Context
[11]	Scanner data	11.1 million transactions, 17 years	German household panel
[16]	Direct observation	462 menu choices, 2 weeks	Fine-dining restaurant, Oslo
[12]	Self-report	7-point Likert scale, 4 items (intention) + 3 items (behavior)	Online survey, Jiangsu, China
[13]	Self-report	5-point Likert scale, intention and behavior measured simultaneously	Online survey, tier-1 cities, China

**Table 19:** Statistics of the Intention–Behavior Relationship.

Study	Coefficient	Behavior Type	Interpretation
[11]	β = 0.022 ***	Scanner data	Virtually null effect
[12]	β = 0.446–0.505 ***	Self-report	Moderate effect
[13]	β = 0.395–0.488 ***	Self-report	Moderate effect, varies by product

Note: *** Asterisk indicate statistical significance levels (*p* < 0.001). Estimated average from the psychometric literature [1,2,4], used as baseline for comparison, as [11] did not measure attitudes directly. The divergence index is calculated as: (self-report − objective)/self-report × 100.

**Table 20:** Methodological Combinations.

Study	Combination	Gap
[13]	Scale + Scanner	Discrete Choice Experiment (DCE)
[16]	Field experiment + Point of Sale (POS)	Scale, Discrete Choice Experiment (DCE)
[12]	Scale + Self-report	Objective behavior, Discrete Choice Experiment (DCE)

Note: The sum of studies across domains exceeds 62 due to studies that address multiple research questions and were coded in more than one category. This overlap reflects the multidimensional nature of sustainable food behavior research and is accounted for in the configurational mapping (Section 2.5).

**Table 21:** Integrated Architecture of Sustainable Food Behavior Research.

Layer/ Domain	Domain & Constructs	Analytical Role & Key References
1. Structural Context (Macro)	Environmental sustainability; Ethical consumption; Cultural context; Institutional trust; Marketing mix; Product availability; Price sensitivity; Motivation, Opportunity, and Ability (MOA)	Influences Perceived Behavioral Control (PBC), trust, and attribute salience; Theory of Planned Behavior (TPB) extensions; Value–Belief-Norm (VBN); external validity studies
2. Motivational Latent Structures	Health motives; Sensory appeal; Natural concerns; Weight control; Convenience; Ethical concern; Environmental values; Moral & social norms; Food values; Sustainability orientation; Transparency/Clean-label; Image-based schemas	Predicts intention or utility (Hybrid Choice Model (HCM)); Food Choice Questionnaire (FCQ); the Eating Motivation Survey (TEMS/TEMS-BR); Sustainable Food Choice Questionnaire (SUS-FCQ); Theory of Planned Behavior (TPB); Value-Belief-Norm (VBN); Clean Food Consumerism (CFC); Image Theory
3. Cognitive-Heuristic Interference	Trust (eco-label, institutional, situational); Skepticism (greenwashing, climate); Halo & affective heuristics; Defaults; Availability; Multi-label effects; Knowledge; Social desirability	Moderates attitude → intention, intention → behavior; trust & bias literature; eco-label studies; behavioral experiments
4. Experimental Market Attributes	Price; Organic; Animal welfare; Local/national origin; Carbon labels; Integrated Pest Management (IPM); Traceability; Multi-level eco-labels; Biocontrol; Gene editing; Irradiation; Antibiotic/pesticide-free	Directly enter utility (standardized regression coefficient (β) attributes); generate Willingness to Pay (WTP); Discrete Choice Experiment (DCE) studies (Multinomial Logit (MNL), Mixed Logit (MXL), Generalized Multinomial Logit (GMNL))
5. Econometric Decision Mechanism	Utility (Uij); Choice probability; Willingness to Pay (WTP); Preference & scale heterogeneity; Class membership; Model specification	Estimates P (choice sustainable) via logit-based probability; Random Utility Theory; Hybrid modeling (Hybrid Choice Model (HCM)/Integrated Choice and Latent Variable (ICLV))
6. Behavioral Outcome Layer	Scanner data; Direct observation; Field experiments; Retrospective self-report; Intention-behavior measures	Tests predictive validity; quantifies intention-behavior decay; external validity studies
7. Validation Architecture	Reliability (Cronbach’s alpha (α), McDonald’s omega (ω)); Convergent (Average Variance Extracted (AVE), Composite Reliability (CR)) & discriminant (Heterotrait-Monotrait Ratio (HTMT)) validity; Structural fit (Comparative Fit Index (CFI), Root Mean Square Error of Approximation (RMSEA)); Measurement invariance; Temporal stability (Intraclass Correlation Coefficient (ICC)); Effect sizes (standardized regression coefficient (β), coefficient of determination (R^2^))	Ensures comparability, robustness, and predictive credibility; COSMIN-adapted criteria; econometric validation standards

## Data Availability

The raw data supporting the conclusions of this article will be made available by the authors on request.

## Supplementary Materials

The following supporting information can be downloaded at: https://www.mdpi.com/article/10.3390/foods15081442/s1, Figure S1: PRISMA; Table S1: LOGBOOK—INTEGRATIVE SYSTEMATIC REVIEW; Table S2: LOGBOOK—DCE SECTION DISCRETE CHOICE EXPERIMENTS; Table S3 Trust, Skepticism & Cognitive Biases Logbook; Table S4: LOGBOOK—REVISÃO SISTEMÁTICA INTEGRATIVA.

## Abbreviations

The following abbreviations are used in this manuscript:

AVE	Average Variance Extracted
BWS	Best-Worst Scaling
CFA	Confirmatory Factor Analysis
CFC	Clean Food Consumerism
CFI	Comparative Fit Index
CR	Composite Reliability
D	Durables
DCE	Discrete Choice Experiment
EIC	Environmental Impact Claims
ELQ	Eco-Label Information Quality
EPC	Ethical Production Claims
ERPI	Extended Reasoned Personal Involvement
ErrVarNorm	Normalized Error Variance
FCQ	Food Choice Questionnaire
FMCG	Fast-Moving Consumer Goods
GMNL	Generalized Multinomial Logit
HBC	Health Benefit Claims
HCM	Hybrid Choice Model
HTMT	Heterotrait-Monotrait Ratio
IAT	Implicit Association Test
ICC	Intraclass Correlation Coefficient
ICLV	Integrated Choice and Latent Variable
IIA	Independence of Irrelevant Alternatives
IPM	Integrated Pest Management
IRT	Item Response Theory
LHS	Labeled Hedonic Scale
MNL	Multinomial Logit
MOA	Motivation, Opportunity, and Ability
MXL	Mixed Logit
NEP	New Ecological Paradigm
PBC	Perceived Behavioral Control
PICOS	Population, Intervention, Comparison, Outcomes, and Study design
POS	Point of Sale
SEM	Structural Equation Modeling
SUS-FCQ	Sustainable Food Choice Questionnaire
TEMS	The Eating Motivation Survey
TEMS-BR	The Eating Motivation Survey-Brazil
TPB	Theory of Planned Behavior
VAB	Value-Attitude-Behavior
VBN	Value-Belief-Norm
WTP	Willingness to Pay

## Appendix A. Full Search Strategy (Web of Science)

The Boolean search string used in Web of Science was:

TS = (

(“Food Choice Questionnaire” OR “FCQ” OR “psychometric*” OR “scale validation”)

AND

(“discrete choice experiment*” OR “DCE” OR “choice model*” OR “willingness to pay” OR “WTP”)

AND

(“sustainable food” OR “organic food” OR “eco-label*” OR “environmental label*” OR “green consumption”)

AND

(“validation” OR “predictive validity” OR “attitude-behavior gap” OR “behavioral intention”)

)

Filters applied:

- Document type: Article
- Language: English
- Time period: 2015–2025

Equivalent adaptations of this query were used for Scopus, PubMed, and PsycINFO, adjusting syntax according to database requirements. Google Scholar searches followed the same conceptual structure but were manually filtered due to platform limitations.
