# Supplementary figures and images for: Measurement and Modeling of Sustainable Food Choice and Purchasing Behavior: A Systematic Review of Methods and Models
Source: Foods. 2026 Apr 21;15(8):1442. doi: 10.3390/foods15081442 (PMC13115353; doi:10.3390/foods15081442)

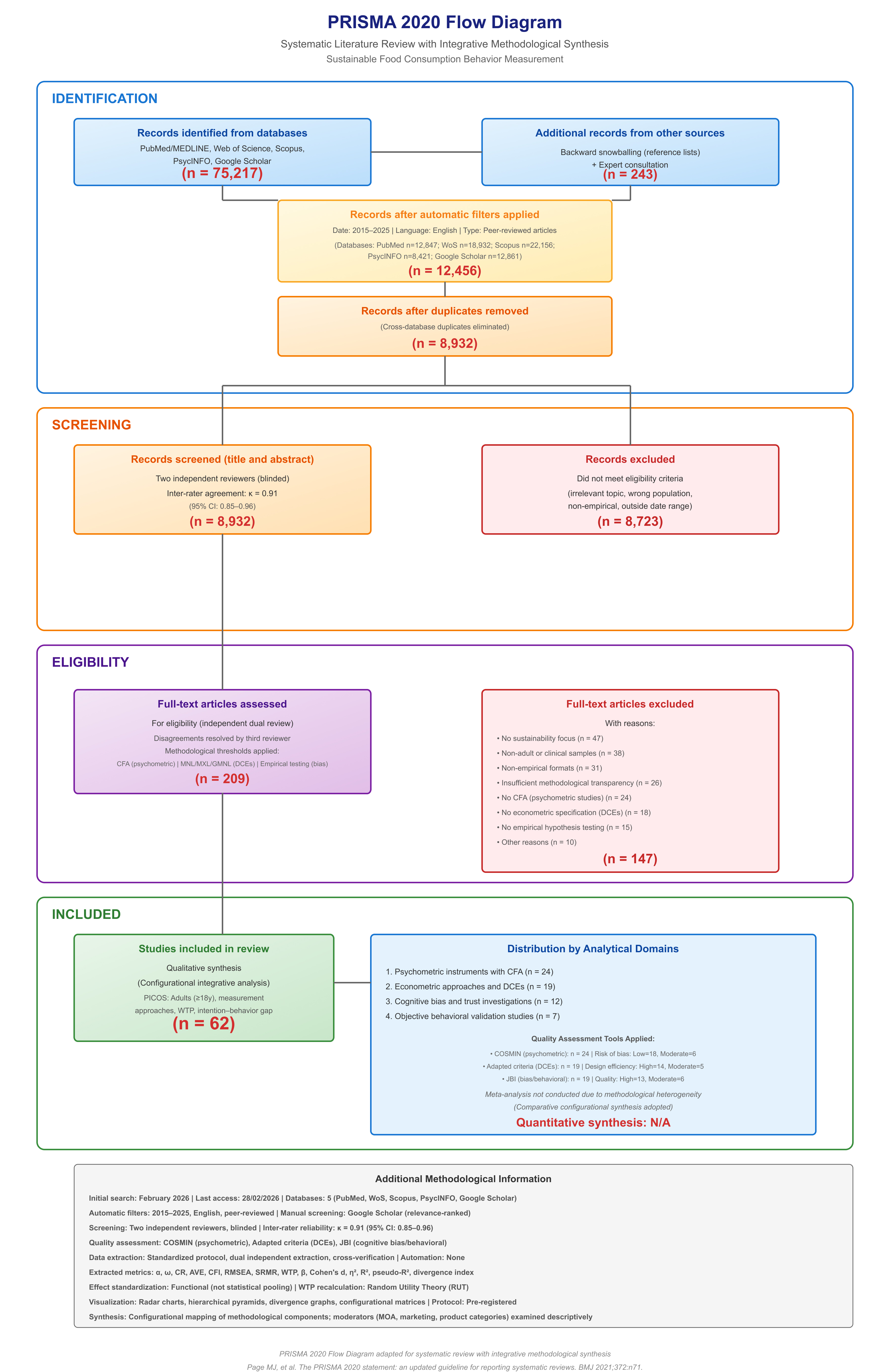

Supplement: Supplementary file 1 [file foods-15-01442-s001.zip › Figure S1 PRISMA.jpg]
